# The effort hypothesis at the heart of the virtuous circle

**DOI:** 10.17179/excli2025-8937

**Published:** 2025-11-12

**Authors:** Michel Audiffren, Nathalie André

**Affiliations:** 1 Centre de Recherches sur la Cognition et l'Apprentissage, Université de Poitiers, CNRS, Poitiers, France

**Keywords:** adherence, brain networks, cognitive control, executive functions, exercise, functional connectivity

## Abstract

This article updates the “virtuous circle” model, which links physical exercise with cognition. This model, which originally focused on connectivity between the salience network (SN) and central executive network (CEN), now also incorporates the default mode network (DMN). It describes a bidirectional dynamic: exercise enhances executive functions (i.e., inhibition, flexibility, updating, planning, and problem-solving), which in turn strengthen long-term exercise adherence. This virtuous circle leads to cognitive, physiological, and motivational benefits through synergistic mechanisms induced by exercise such as the *effort hypothesis* (effort as an investment), the neurotrophic hypothesis, the cardiovascular hypothesis, the inflammatory hypothesis and the glucocorticoid hypothesis. These mechanisms improve connectivity within large-scale neuronal networks, thereby consolidating behavioral regulation. Compared with other behavior change models (e.g., regulation, dual-process, stage-based, and integrative models), the virtuous circle model is notable in light of its circular nature and emphasis on sustainability. In this theoretical framework, adherence to exercise is defined as an evolving strength of the attitude-behavior link, which is shaped by three interconnected processes: immediate motivation (pleasure, mood improvement, social interaction, and rewards), which initiates engagement; sustained effort, which enhances executive control, reduces perceived costs, and fosters habit formation; and behavior-driven attitude change, through cognitive dissonance and effort justification, which aligns beliefs with actions. Recent longitudinal studies have supported the reciprocal associations among exercise, cognition, and brain health, although further trials are needed. This model highlights the fact that early adoption of the virtuous circle promotes the development of health-protective habits, thereby slowing both physical and cognitive aging. In contrast, sedentary lifestyles foster a vicious circle that accelerates decline.

See also the graphical abstract(Fig. 1).

## 1. Introduction

Since its publication in 2019, the seminal paper presenting the virtuous circle model linking exercise and cognition (Audiffren and André, 2019[7]) has received increasing attention, with 75 citations in indexed journals published in English (source: Web of Science 2025). Notably, 20 of these references highlight the value of the virtuous circle model as a key theoretical framework supporting the bidirectional relationship between exercise and cognitive control (e.g., Ludyga et al., 2021[119]; Oliveira et al., 2022[139]; Zhou et al., 2023[205]; Zhao et al., 2024[203]; Gerber et al., 2025[73]). In contrast, many other studies have expanded on these insights by considering only the unidirectional causal link from exercise to cognition (e.g., Chen et al., 2020[32]; Honma et al., 2021[89]; Dai et al., 2024[42]). Finally, other studies have referred to the virtuous circle model by focusing on the relationship between self-control and healthy behaviors, such as regular exercise (e.g., Pesce et al., 2021[144]; Schmengler et al., 2022[162]; Maltagliati et al., 2024[123]).

**Figure 1:**
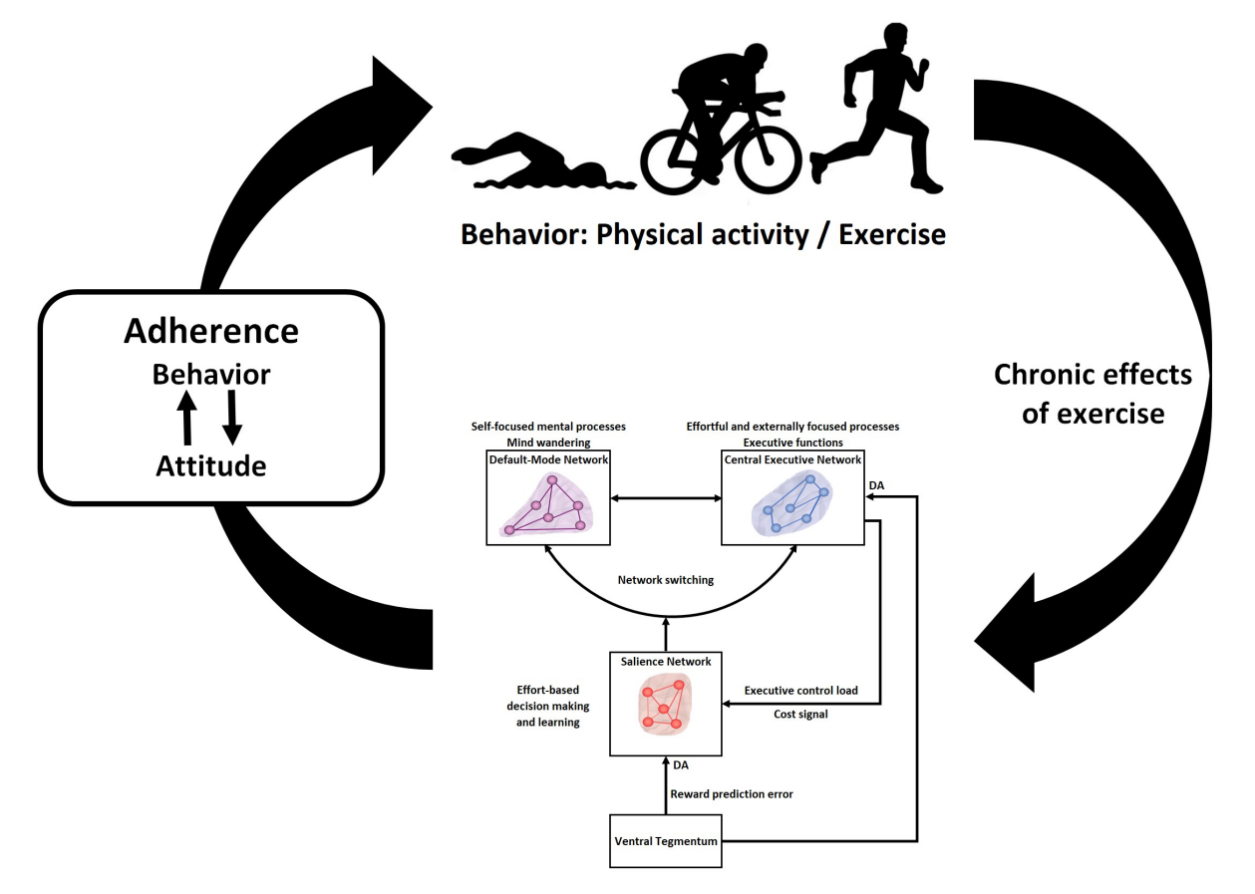
Graphical abstract: The virtuous circle linking exercise and cognition. The right side of the virtuous circle indicates the causal link between chronic exercise and changes in connectivity within and between three large-scale neuronal networks involved in cognition: the central executive network, the default-mode network, and the salience network. These changes in connectivity lead to an increase in willpower, i.e., the capacity to maintain cognitive control despite of high costs. The left side of the virtuous circle indicates the causal link between changes in connectivity that lead to an increase in willpower and adherence to exercise. The arrows linking behavior (exercising) and attitude (beliefs about exercise) illustrate the strength of the relationship between these two variables with respect to exercise adherence.

The virtuous circle model consists of these two causal relationships and assumes a dynamic and bidirectional relationship between behavior (i.e., exercising) and the brain functions associated with cognitive control and executive functions (see Figure 1(Fig. 1)). Cognitive control is defined as a set of cognitive processes that regulate behavior when automatic routines are not available to cope with the situation or are not appropriate for reaching the planned goal (Pessiglione et al., 2025[145]). Executive functions (EFs) are a set of higher-order cognitive abilities that operationalize cognitive control. They include processes such as working memory, inhibitory control, cognitive flexibility, planning, and problem-solving (Diamond, 2013[45]). The link between cognitive control and EFs is hierarchical and functional: Cognitive control sets the goals and priorities to cope with the situation, while EFs implement the strategies necessary to achieve them.

The *effort hypothesis* describes one mechanism that underlies and maintains the virtuous circle. It implies, on the one hand, the mechanisms that mediate the causal relationship between exercise and three large-scale neuronal networks regulating cognitive control and, on the other hand, the mechanisms supporting the causal relationship between gains in cognitive control and the maintenance of exercise over time. Overall, the *effort* *hypothesis* assumes that maintaining the dynamic relationship between exercise and cognitive control changes the connectivity within and between three large-scale neuronal networks involved in cognition in such a way that the ability to exert cognitive control is enhanced and facilitates the induction and maintenance of behavior. These three large-scale neuronal networks are presented in section 5.

The principal aim of the present paper is to update our knowledge of the mechanisms involved in the *effort hypothesis* in light of the recent advances made in the fields of social psychology, cognitive psychology and neuroscience. A secondary objective of this paper is to update the model by considering the role of self-referential thoughts in the capacity to sustain cognitive control during a cognitive or physical task (see section 5) and in the capacity to sustain regular and long-term engagement in physical activity through attitudes (see section 8.3). Self-referential thoughts are mental processes in which attention and reflection are directed toward oneself. They notably include the evaluation of one's own emotions, beliefs, experiences, and intentions; the recollection of personal events (autobiographical memory); the projection of oneself into the future (prospection, imagining scenarios); and the construction and maintenance of the sense of identity or “self.”

## 2. The Virtues of Effort

The *effort hypothesis* is one mechanism that maintains the virtuous circle by linking exercise to improved functional connectivity in the brain, increasing cognitive control and maintaining behavior. To understand and adequately use the *effort hypothesis* within the virtuous circle model, clarification on how effort is conceptualized is needed. Effort has often been viewed as inherently aversive, reducing the appeal of rewards or desirable goals (Hull, 1943[95]; Lewis, 1965[114]). Today, many theories postulate that effort is inherently (or biologically) aversive or unpleasant (e.g., Kool et al., 2010[105]; Inzlicht et al., 2014[98]; Cheval and Boisgontier, 2021[33]) and that benefits or rewards must be sufficiently enough to overcome effort aversion. However, empirical evidence does not show that individuals are disinclined to exert effort (i.e., that they are inherently lazy), but that it is the assessment of costs versus benefits that governs engagement in effortful tasks. Individuals are likely to maintain effort as long as the outcomes justify the expenditure, in accordance with the principles of least effort and optimization of resources (Gendolla et al., 2012[72], 2019[71]). Therefore, the *effort hypothesis* postulates that readiness to engage in effort is determined by cost-benefit analysis (or computation) rather than the capacity to overcome the unpleasantness of effort. The consequence is that people naturally try to avoid exerting effort that seems unnecessary or wasted, not to avoid exerting effort itself. In this way, the brain constantly evaluates costs and benefits related to effort exertion to manage resources efficiently and maintain allostasis, i.e., the process by which the organism anticipates, adapts, and dynamically adjusts its physiological systems, such as the nervous, endocrine, and immune systems, in response to stressors and environmental challenges (McEwen and Wingfield, 2003[127]). This perspective highlights an important virtue of effort: it represents a rational investment of resources directed toward meaningful outcomes rather than wasted energy. The *effort hypothesis* suggests that encouraging people to value effort requires highlighting situations where the rewards of investing energy clearly outweigh the costs.

Effort can be positively valued when it is associated with meaningful outcomes, such as skill acquisition, physical fitness, or personal satisfaction. Laboratory studies show that exerting effort is often experienced as neutral or even positive (Clay et al., 2022[35]), illustrating that effort is both costly and rewarding (e.g., Inzlicht et al., 2018[99]). Motivational intensity theory further supports the claim that effort expenditure aligns with perceived outcome value (Brehm and Self, 1989[22]; Gendolla et al., 2012[72], 2019[71]). In this way, effort exertion can be seen as a pathway to growth, achievement, and fulfillment, virtues that emerge precisely because energy is invested rather than withheld. Reconceptualizing effort as costly rather than inherently aversive frames humans and many animals as rational agents who make optimized decisions: effort is avoided when unjustified, but willingly undertaken when the expected benefits outweigh the costs.

## 3. Foundations of the Virtuous Circle Model

The virtuous circle model builds on the following broad stream of research. Cross-sectional, longitudinal, and epidemiological studies have demonstrated associations between physical activity and cognition (e.g., Rockwood and Middleton, 2007[157]; Engeroff et al., 2018[52]). Complementary evidence from randomized controlled trials (RCTs) has validated the effects of chronic exercise on EFs and on the brain regions involved in effort-based decision making (e.g., Colcombe and Kramer, 2003[36]; Colcombe et al., 2006[37]). Longitudinal work further highlights the role of self-control in shaping health behavior (e.g., Moffitt et al., 2011[134]; Fergusson et al., 2013[63]; Richmond-Rakerd et al., 2021[156]). Finally, RCTs indicate that improvements in executive functioning, self-control, and effortful control enhance adherence to exercise (e.g., McAuley et al., 2011[125]; Best et al., 2014[12]; Savikangas et al., 2021[161]).

The selective causal relationship between exercise and EFs was hypothesized more than twenty years ago by Arthur Kramer and his collaborators through the *selective improvement hypothesis* (Kramer et al., 1999[106]), which was subsequently renamed the *executive-control hypothesis* (Colcombe and Kramer, 2003[36]). The *selective improvement hypothesis* proposed that exercise leads to selective improvement in tasks including a substantial frontal-lobe-dependent executive control component, rather than generalized benefits in all domains of cognition. More precisely, according to these authors, tasks requiring executive control exhibit the greatest aging-related declines and, simultaneously, show the greatest improvements in performance as a result of exercise.

Two main distinctions separate the *selective improvement hypothesis* from the *effort hypothesis*. First, the *selective improvement hypothesis* focuses only on the right part of the virtuous circle, i.e., the effects of chronic effects of exercise on executive control, whereas the *effort hypothesis* embraces the whole virtuous circle; i.e., it also includes the mechanisms explaining that long-term changes in connectivity within and between these three large-scale neuronal networks caused by exercise determine our future health behaviors (see Figure 1(Fig. 1)). Second, as mentioned, the *selective improvement hypothesis* focuses only on executive control, whereas the *effort hypothesis* includes more broadly the chronic effects of exercise on the connectivity within and between three large-scale neuronal networks involved in executive control, effort-based decision making and internally focused thoughts.

## 4. Chronic Effects of Exercise on Cognitive and Brain Health

**Figure 2:**
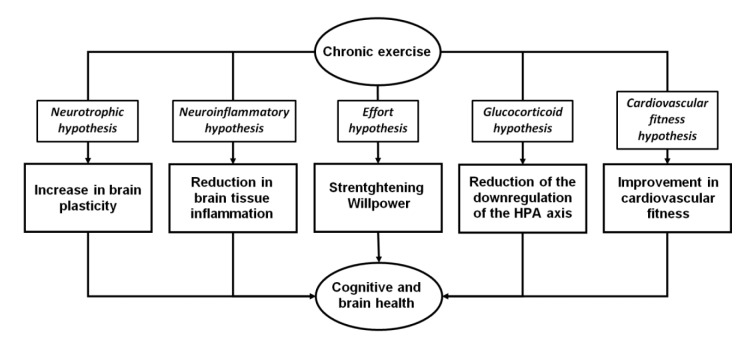
The different mechanisms mediating the chronic effects of exercise on cognitive and brain health.

The causal link between chronic exercise and cognitive health has been clearly established in children, middle-aged adults, and aging people through numerous meta-analyses of randomized controlled trials (e.g., Falck et al., 2019[58]; Sanders et al., 2019[159]; Xue et al., 2019[198]; Zhang et al., 2023[201]; Akalp et al., 2024[3]; Ye et al., 2024[199]; Singh et al., 2025[168]). Five main plausible biological mechanisms leading to the benefits of chronic exercise on brain health have been identified (see Figure 2(Fig. 2)).

The *cardiovascular fitness hypothesis* suggests that cardiovascular adaptations to aerobic exercise (i.e., a gain in the efficiency of the respiratory and circulatory systems at providing oxygenated blood to relevant musculature during active moments) ameliorate cerebral blood flow, brain perfusion, and brain oxygenation and, consequently, cognitive and brain functions (e.g., Pentikäinen et al., 2017[143]; Thomas et al., 2020[181]). The mediating effect of cardiovascular fitness on the relationship between aerobic exercise and cognition has been tested several times in RCTs and the results are inconclusive (e.g., Etnier et al., 2006[55]; Smiley-Oyen et al., 2008[170]; Albinet et al., 2016[4]; Billinger et al., 2017[14]), whereas the results of cross-sectional and longitudinal studies are more convincing (e.g., Barnes et al., 2003[10]; Aberg et al., 2009[1]; Vidoni et al., 2012[185]; Boots et al., 2015[18]). Additional intervention studies are needed to conclusively demonstrate the contribution of this mechanism to the positive effects of exercise on cognition.

The *glucocorticoid hypothesis* posits a progressive downregulation of the hypothalamo-pituitary-adrenal (HPA) axis with aging (Sapolsky et al., 1986[160]; Garrido, 2011[69]). Stress activates the HPA axis, beginning with corticotrophin-releasing hormone (CRH) secretion from the hypothalamus, followed by adrenocorticotropic hormone (ACTH) release from the pituitary, and subsequent glucocorticoid (cortisol) secretion from the adrenal cortex. Glucocorticoids mobilize energy and provide inhibitory feedback on CRH and ACTH release (Sapolsky et al., 1986[160]). Chronic stress and aging may impair this feedback, causing HPA hyperactivity and elevated cortisol levels. A second assumption in this context is that chronic exercise mitigates these detrimental effects (Chen et al., 2017[31]), either by buffering cortisol secretion (Lalanza et al., 2012[109]; Hare et al., 2014[82]) or by upregulating medial prefrontal dopamine, which enhances negative feedback of the HPA axis (Diorio et al., 1993[48]; Herman et al., 2003[84]). Moreover, chronic stress, but not exercise, downregulates mineralocorticoid and glucocorticoid receptors in the hippocampus (McEwen et al., 1968[126]; Sapolsky et al., 1986[160]) and impairs adult neurogenesis in the dentate gyrus (Schoenfeld and Gould, 2012[163]). While the *glucocorticoid hypothesis* is plausible, it remains to be fully validated in humans.

The *neurotrophic hypothesis* proposes that chronic exercise stimulates the release of neurotrophic factors that support brain plasticity through hippocampal neurogenesis, angiogenesis, and long-term potentiation (LTP). Exercise promotes neuronal proliferation, differentiation, and integration in the dentate gyrus (Brown et al., 2003[24]; van Praag, 2008[184]; Itoh et al., 2011[100]; Farioli-Vecchioli and Tirone, 2015[59]; Liu and Nusslock, 2018[115]; El-Sayes et al., 2019[51]; Voss et al., 2019[189]; Lambertus et al., 2024[110]). It also enhances brain angiogenesis, as documented in rodent treadmill studies (Stevenson et al., 2020[174]; Zang et al., 2023[200]; Maheu et al., 2025[122]). LTP, a cellular mechanism underlying learning and memory (Paoletti et al., 2013[141]), is facilitated by chronic exercise (Radahmadi et al., 2016[153]; Moore and Loprinzi, 2020[136]; Vints et al., 2022[186]). Neurotrophic factors such as brain-derived neurotrophic factor (BDNF), insulin-like growth factor-1 (IGF-1), and vascular endothelial-derived growth factor (VEGF) mediate these effects (Cotman et al., 2007[40]; Voss et al., 2013[187]; Canivet et al., 2015[26]; Canivet et al., 2017[27]). BDNF, which is primarily synthesized in glutamatergic neurons, is central to exercise-dependent neuroplasticity, neurogenesis, and LTP (Poo, 2001[149]; Binder and Scharfman, 2004[15]; Lu et al., 2014[118]), whereas IGF-1 and VEGF increase peripherally during exercise, cross the blood-brain barrier, and stimulate neurogenesis and angiogenesis (Trejo et al., 2001[183]; Fabel et al., 2003[57]; Lopez-Lopez et al., 2004[117]). Overall, neurotrophic factors are critical to learning, memory, and cognitive health (Bibel and Barde, 2000[13]; Huang and Reichardt, 2001[93]) and have received strong support from animal studies, although only indirect evidence in humans has been reported (Erickson et al., 2011[53]).

The *neuroinflammatory hypothesis* posits that aging induces changes in both the central nervous system (CNS) and peripheral immune cells, resulting in chronic low-grade inflammation, characterized by elevated proinflammatory cytokines and linked to neurodegenerative diseases (Bauer et al., 2024[11]). In the aging brain, microglia adopt a proinflammatory phenotype, releasing cytokines such as TNF-α and facilitating T-cell infiltration, thereby sustaining neuroinflammation and cognitive decline (Bauer et al., 2024[11]). Systemic inflammation further exacerbates this process, as cytokines cross the blood-brain barrier or signal via the vagus nerve, activating glial cells and promoting pathogenic events such as beta-amyloid aggregation and tau phosphorylation, key features of Alzheimer's disease. Thus, aging is tightly associated with neuroinflammation arising from both CNS and systemic changes. Conversely, regular exercise exerts anti-neuroinflammatory effects through multiple mechanisms. It shifts microglia toward an anti-inflammatory state (Dimitrov et al., 2017[47]; Chauquet et al., 2024[30]), elevates beneficial cytokines and metabolites such as IL-6, which exert anti-inflammatory effects during exercise (Pedersen et al., 2001[142]; Febbraio et al., 2002[61]; Petersen and Pedersen, 2005[146]; Han et al., 2023[81]), and strengthens the blood-brain barrier, limiting peripheral immune infiltration (Hu et al., 2024[92]). Collectively, physical activity emerges as a potent strategy to counteract age-related neuroinflammation, a hypothesis that is strongly supported by animal studies, although further validation in humans is needed.

The *effort hypothesis* assumes that practicing regular exercise selectively activates neural circuits involved in effort-based decision-making and, through repetition, strengthens connectivity within three large-scale neuronal networks in a specific way, facilitating the deployment of effort despite situational constraints. In this article, effort is defined as the mechanism through which the brain makes decisions about the resources that need to be mobilized to reach a goal, including energy and cognitive control (Sanders, 1983[158]; Hockey, 1997[85]; Gendolla and Wright, 2009[70]; André et al., 2019[5]). Overall, the *effort hypothesis* describes the mechanisms initiating the dynamics of the virtuous circle. It includes three interrelated assumptions: (1) regularly practicing effortful physical exercises associated with intrinsic and/or extrinsic rewards leads to an improvement in the ability to exert executive control and, more generally, to exert efforts to reach a goal despite costs such as fatigue and pain often linked to exercise; (2) these improvements in the cognitive domain are underpinned by long-term changes in connectivity within and between three large-scale neuronal networks involved in cognition and described later in this article; and (3) these improvements in executive and effortful control lead to a facilitation of stopping unhealthy behaviors (e.g., smoking) and adhering to healthy behaviors (e.g., exercising). The following section describes each of these three assumptions more precisely.

The abovementioned five hypotheses can be synergistic, and all of them participate in the maintenance of the virtuous circle. For instance, increased brain plasticity induced by chronic exercise may facilitate changes in connectivity within and between the three large-scale neuronal networks involved in executive functions, effort-based decision making and self-referential thoughts. Similarly, better cardiovascular fitness may decrease the costs of exercise and subsequently facilitate the engagement of effort.

## 5. The Salience Network in the Heart of the Effort Hypothesis

The *effort hypothesis* is based on a large-scale brain network framework, called the triple-network model (Menon, 2011[130]), which describes how three core intrinsic connectivity networks interact to support cognition, emotion, and behavior. It focuses on the dynamic switching between these networks to enable adaptive responses to environmental and internal demands. The three core networks involved in the triple-network model include (see Figure 1(Fig. 1)) the salience network (SN), central executive network (CEN) and default mode network (DMN). The first two networks are clearly activated during tasks that require attention to external stimuli (i.e., task-positive networks), whereas the DMN exhibits reduced activity during the same tasks (i.e., the task-negative network).

The SN refers to a set of brain regions featuring two principal cortical hubs: the dorsal anterior cingulate cortex (dACC) and the ventral anterior insula (vAI). This network was discovered by William Seeley from the University of California (Seeley et al., 2007[165]). Its main functions include detecting and filtering salient internal and external stimuli, initiating switching between the other two networks, and allocating attentional and cognitive resources according to the stimulus relevance. The vAI plays a central role in detecting salient events in a bottom-up manner and in orchestrating switches between the DMN and CEN to optimize access to attentional and working memory resources (Menon, 2023[129]). Evidence across diverse tasks identifies the vAI as a causal signaling hub, promoting activation of the CEN while suppressing activity within DMN regions (Sridharan et al., 2008[172]; Moran et al., 2013[137]; Manoliu et al., 2014[124]; Chand and Dhamala, 2016[29]). In other respects, converging evidence indicates that the dACC integrates the costs and benefits of an ongoing task to determine the level of cognitive control and energy allocated to its completion (e.g., Kennerley et al., 2006[102]; Shenhav et al., 2013[167]; Klein-Flügge et al., 2016[104]).

The CEN, also known as the frontoparietal network, is anchored mainly in the dorsolateral prefrontal cortex (DLPFC) and posterior parietal cortex (PPC). Its primary role is to support high-level cognitive functions called executive functions, which include working memory, inhibitory control, cognitive flexibility, planning, problem-solving, and reasoning (Diamond, 2013[45]).

The DMN is a collection of distributed and interconnected brain regions, including the medial prefrontal cortex (MPFC), posterior cingulate cortex (PCC), and angular gyrus (Menon, 2023[129]). The DMN is generally deactivated when attention is directed toward external stimuli, but becomes active in their absence, supporting internally oriented processes such as self-reflection, mind wandering, daydreaming, recalling personal memories, and imagining the future. Initially identified through its reduced activity during externally focused, cognitively demanding tasks rather than through task-related activation, the DMN is linked to attentional lapses and difficulties disengaging from internal thoughts. It has been widely recognized as supporting five key cognitive domains: self-referential thinking, social cognition, episodic memory, language and semantic memory, and mind wandering (Menon, 2023[129]).

According to the triple-network model, the SN detects external events that are behaviorally relevant. This detection triggers a suppression of the DMN and alters its temporal interactions with cognitive control systems, including the SN itself, the CEN, and the dorsal attention network (DAN). These dynamic changes enable focused attention and working memory processes, which in turn support goal-directed behavior (Menon, 2023[129]). The DAN is a large-scale task-positive brain network that plays a central role in top-down, goal-directed attentional control (Corbetta and Shulman, 2002[39]; Fox et al., 2006[66]). It enables the voluntary allocation of attention to relevant sensory information and the selection of spatial or feature-based targets. The network is bilaterally organized and primarily involves two key hubs: the intraparietal sulcus/superior parietal lobule (IPS/SPL) in the posterior parietal cortex and the frontal eye field (FEF) in the frontal cortex. Together, these regions coordinate attentional orientation, visual search, and the modulation of sensory processing in early visual cortices.

The distinction between task-positive and task-negative networks should be interpreted with caution, as it has been observed, for example, that the PCC, a major hub of the DMN, can be activated during a cognitive task, reflecting learning processes, retrieval of prior knowledge, internal context setting, and the integration of new information, which helps configure brain networks for efficient task execution (e.g., Spreng et al., 2010[171]; Small et al., 2003[169]). These features challenge the oversimplified view of the DMN as purely “task-negative” and highlights its flexible role depending on cognitive demands.

Functional magnetic resonance imaging (fMRI) studies have indicated that attentional lapses are linked to increased activity within the DMN alongside reduced activation of the CEN. For example, Fassbender et al. (2009[60]) reported that greater variability in reaction time correlates with a failure to deactivate the ventromedial prefrontal cortex (VMPFC), a key DMN region, as the task difficulty increases. Similarly, sustained attention deficits in individuals with traumatic brain injury have been associated with elevated DMN activation (Bonnelle et al., 2011[17]). Mind wandering has also been connected to increased DMN activity and diminished CEN engagement (Esposito et al., 2014[54]; Gergelyfi et al., 2021[74]). Complementing these findings, Weber et al. (2022[193]) demonstrated that DMN activation is stronger during periods of low effort than during periods of high effort, thus indicating that DMN activity is dynamically modulated according to effort demands. All these studies clearly show that inappropriate activation of the DMN during the execution of a cognitive task is associated with difficulties in maintaining focus, that is, in effectively engaging EFs. Inappropriate DMN activation occurs in cases such as mental fatigue, aging, psychopathologies (e.g., ADHD, schizophrenia), and nervous system injuries (e.g., traumatic brain injury, Alzheimer's disease). According to the *effort hypothesis*, the regular practice of physical exercises that require mental effort deployment can, in contrast, help develop willpower, the ability to exert mental effort and cognitive control despite adverse conditions (e.g., fatigue, extreme environments, likely failure); in other words, it can improve the regulation of DMN deactivation when it is needed for task performance.

The SN is believed to play a key role in the self-regulatory aspects of physical performance, rather than in its purely motor components. Specifically, during exercises that require stamina, the SN, particularly the dACC, is believed to regulate the willingness to initiate and sustain goal-directed physical effort (Winterer et al., 2002[196]). The *effort hypothesis* posits that exercise training requiring sustained mental effort induces long-term changes in brain connectivity, both within and between the three networks of the triple-network model, which enhance the capacity to maintain performance despite increasing feelings of fatigue, boredom, and pain. More precisely, exercise training may stabilize a motivational context essential for maintaining goal-directed action sequences through reinforcement learning (Holroyd and Yeung, 2012[88]), particularly within the SN.

## 6. Empirical Evidence Supporting Functional Connectivity Changes with Physical Training

**Figure 3:**
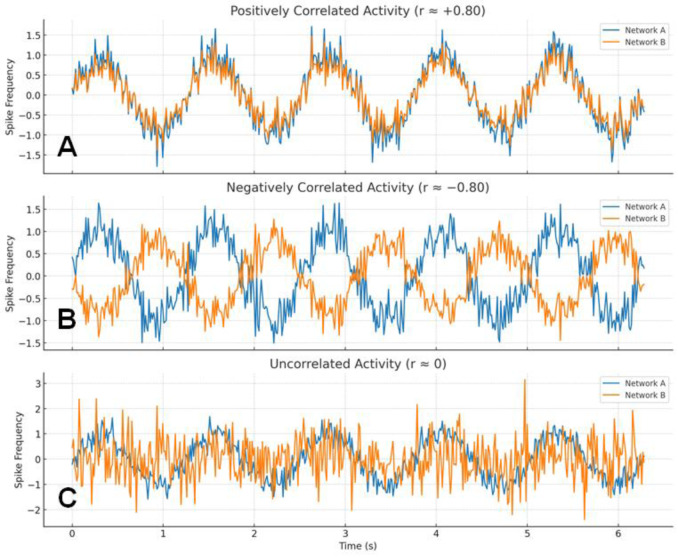
The three patterns of correlation between two brain regions belonging to two brain networks A and B. A: Positive correlation = activities of networks A and B are synchronized. B: negative correlation = activities of networks A and B are in the opposite phase. C: no correlation = activities of networks A and B are dissociated and independent.

In fMRI, functional connectivity (FC) refers to the statistical dependence between blood oxygenation level-dependent (BOLD) signal time series from distinct brain regions. Regions that show correlated activity over time are considered functionally connected, regardless of direct structural links. When the activity of two brain regions is positively correlated, their activities are synchronized and the FC is high (see Figure 3A(Fig. 3)). In contrast, when the activity of two brain regions is inversely correlated (i.e., negatively correlated), their activities are in the opposite phase and indicate an inverse functional relationship, thus suggesting that these regions may participate in brain networks with antagonistic functions (e.g., the DMN vs. the CEN) (see Figure 3B(Fig. 3)). Finally, when the activity between two brain regions is not correlated, no consistent temporal relationship is evident (see Figure 3C(Fig. 3)); when one region's activity increases or decreases, the other does not show a predictable change. In other words, the two brain regions are considered functionally disconnected.

Resting-state FC (rs-FC) measures spontaneous correlations in brain activity when no explicit task is performed, whereas task-based FC measures correlations that occur specifically during the performance of a cognitive or motor task. Within-network FC refers to the spontaneous correlation between the activity of a pair of brain regions belonging to the same network (e.g., the DLFC and PPC of the CEN), whereas between-network FC refers to the spontaneous correlation between two brain regions belonging to two different networks (e.g., the vAI of the SN and the PCC of the DMN).

The *effort hypothesis* posits that regular physical training induces long-lasting modifications in brain network connectivity, particularly by strengthening the functional integration and dynamic interactions among the SN, DMN, and CEN. These neural adaptations underlie enhanced willpower, cognitive resilience, sustained attention, and executive control, supporting the concept that exercise is a potent intervention for maintaining and improving brain health across the lifespan. More precisely, the *effort hypothesis* predicts a higher rs-FC with exercise training within and between task-positive networks, such as the SN, CEN, and DAN, and, to a lesser extent, within the DMN. With respect to changes in rs-FC between task-positive networks and the DMN, the pattern of results is more difficult to predict, as the DMN comprises a very large set of brain regions, some of which can be activated during cognitive tasks. For such changes in inter-network connectivity between task-positive and task-negative networks, region-to-region predictions would need to be established, a task that lies beyond the scope of this article.

In this section, we aim to provide arguments in support of the *effort hypothesis* on the basis of cross-sectional, longitudinal, and interventional studies that have examined the effects of regular physical activity practice on rs-FC between brain regions of interest. The studies reported in this section were identified from four review articles (Bray et al., 2021[21]; Won et al., 2021[197]; Huang et al., 2022[94]; Moore et al., 2022[135]) and a bibliographic search. Only studies including regions of interest of the SN, CEN, DAN and DMN are presented.

Eight cross-sectional studies and two longitudinal studies using resting-state fMRI that satisfied our inclusion criteria were identified. The characteristics and main results of these studies are summarized in Table 1(Tab. 1) (References in Table 1: Boraxbekk et al., 2016[19]; Boyne et al., 2018; Dorsman et al., 2020[50]; Gust et al., 2022[78]; Ikuta and Loprinzi, 2019[97]; Ikuta et al., 2019[96]; Liu et al., 2018[116]; Peven et al., 2019[147]; Raichlen et al., 2016[154]; Stillman et al., 2018[175]; Voss et al., 2016[191]). None of these studies specifically tested the *effort hypothesis*, and all of the results presented in Table 1(Tab. 1) are inconsistent and cannot be used to support or refute the *effort hypothesis*. Only the study by Raichlen et al. (2016[154]) clearly supports the *effort hypothesis* concerning task-positive networks and shows that endurance athletes have higher within-network connectivity within the CEN than non-athletes. Three other studies support higher within-network functional connectivity within the DMN in individuals with higher cardiorespiratory fitness (Boraxbekk et al., 2016[19]; Ikuta and Loprinzi, 2019[97]; Ikuta et al., 2019[96]).

In other respects, thirty-seven interventional studies measuring rs-FC and including a physical component in the intervention have been identified. The characteristics of these studies are presented in Supplementary Information, S1. In this section, we discuss only the results from the 19 RCTs examining the changes in rs-FC induced by exercise within and between the SN, CEN, DAN, and DMN. As in the case of the cross-sectional studies, none of the interventional studies specifically tested the *effort hypothesis*.

The theoretical framework of most RCTs reviewed in this section is based on the broad assumption that exercise, or the combination of physical and cognitive training, stimulates brain plasticity, thereby driving changes in functional connectivity (see Supplementary Information, S2). Only three studies proposed more specific hypotheses regarding networks involved in cognitive control (Hsu et al., 2017[91]; Tao et al., 2017[179]; Prehn et al., 2019[151]). Five others focused on the hippocampus and the DMN in relation to memory (Burdette et al., 2010[25]; Eyre et al., 2016[56]; Tao et al., 2016[180]; Flodin et al., 2017[65]; Broadhouse et al., 2020[23]). Finally, four RCTs targeted both the DMN and networks associated with cognitive control (Voss et al., 2010[188]; Pieramico et al., 2012[148]; Suo et al., 2016[178]; Voss et al., 2019[190]). Notably, 79 % of the RCTs were conducted in older adult samples.

Among the 19 RCTs identified in this context, only four supported the *effort hypothesis*, showing strengthened functional connectivity within the SN (Voss et al., 2019[190]; Leocadi et al., 2024[112]), within the CEN (Voss et al., 2010[188]), and between the SN and CEN (Balazova et al., 2021[9]). Interestingly, the latter study also demonstrated that this connectivity change correlated with improvements in executive task performance.

Seven studies reported increased connectivity within the DMN (Voss et al., 2010[188]; Pieramico et al., 2012[148]; Eyre et al., 2016[56]; Tao et al., 2016[180]; Broadhouse et al., 2020[23]; Balazova et al., 2021[9]; Zhu et al., 2021[206]). In two of these studies, changes in DMN connectivity were associated with increased memory performance (Pieramico et al., 2012[148]; Eyre et al., 2016[56]), whereas in two others, they were linked to improvements in EFs (Voss et al., 2010[188]; Zhu et al., 2021[206]).

One study revealed no evidence of connectivity changes after six weeks of balance training (Magon et al., 2016[121]). In contrast, three studies reported results inconsistent with previous findings, showing decreased functional connectivity within the DMN (Tozzi et al., 2016[182]; Dimitriadis et al., 2024[46]) and the DAN (Hsu et al., 2017[91]). These interventions consisted of aerobic exercise programs lasting 16, 12, and 24 weeks, respectively.

Finally, four RCTs reported changes in connectivity between the DMN and the CEN, with four showing increases (Suo et al., 2016[178]; Flodin et al., 2017[65]; Prehn et al., 2019[151]; Claus et al., 2023[34]). However, the study by Claus et al. (2023[34]) also reported a decrease. Notably, the increase reported by Suo et al. (2016[178]) was induced by cognitive training and correlated with improvements in memory performance.

Current evidence suggests that regular physical activity can modulate functional connectivity within brain networks, notably the SN, CEN, DAN, and, to a lesser extent, the DMN. Thus, while the *effort hypothesis* is partially supported, future research using standardized protocols and targeted analyses of specific networks is needed to robustly establish the impact of exercise on brain connectivity.

## 7. Executive Functions (EFs) as Core Processes of Behavioral Change Models

Behavior change and the long-term maintenance of a health behavior are essential components of the virtuous circle model. The purpose of this section is to highlight the role of EFs in this process of behavior change by examining how EFs have been integrated into different behavior change models, and how the virtuous circle model differs from them.

Since the 1980s, EFs have been introduced in theoretical models dedicated to behavioral changes. This new generation of models started with regulatory models with the goal of narrowing the intention-action gap (Leventhal et al., 1980[113]; Kuhl and Beckman, 1985[108]; Heckhausen and Gollwitzer, 1987[83]; Kruglanski et al., 1990[107]; Gollwitzer, 1999[75]). Regulatory theories have focused on complex and multidimensional processes that cannot be reduced to mere motivation or sheer willpower. Some models emphasize the central role of planning (Gollwitzer's Implementation Intention; Heckhausen and Gollwitzer's Rubicon Model), which enables individuals to transform intentions into concrete plans and to cross the threshold from wanting to acting. Others highlight the importance of sustaining action over time through attention and monitoring (Kuhl and Beckmann's Action Control Theory), or the ability to adapt when facing obstacles and unexpected challenges (Leventhal and collaborators' Commonsense Model). Finally, Kruglanski's goal systems theory reminds us that goals do not operate in isolation but within a dynamic network of associations, activations, and inhibitions. Taken together, these models suggest that EFs rely on an interplay of preparation, persistence, and flexibility, involving not only the initiation of action but also the maintenance of effort and adaptation to both internal and external constraints (see Table 2(Tab. 2); References in Table 2: Audiffren and André, 2019[7]; Brand and Ekkekakis, 2017[20]; De Vries, 2017[44]; Gollwitzer, 1999[75]; Hagger and Chatzisarantis, 2014[79]; Hall and Fong, 2007[80]; Heckhausen and Gollwitzer, 1987[83]; Hofmann, Friese and Strack, 2009[87]; Kruglanski et al., 1990[107]; Kuhl and Beckmann, 1985[108]; Leventhal et al., 1980[113]; Michie, van Stralen et West, 2011[132]; Rhodes, 2017[155]; Schwarzer and Luszczynska, 2008[164]; Strack and Deutsch, 2004[176]; Strobach et al., 2020[177]; Weinstein, Sandman and Blalock, 2008[195]).

Dual-process models highlight the interaction between reflective and impulsive systems in guiding behavior. The Physical Activity Adoption and Maintenance Model (PAAM) emphasizes the role of planning and self-regulation in sustaining long-term behaviors such as physical activity (Strobach et al., 2020[177]). The Affective-Reflective Theory adds that self-control depends on the interplay between immediate affective reactions and reflective evaluations, showing how emotions shape decisions about engaging in or avoiding certain behaviors (Brand and Ekkekakis, 2018[20]). The Reflective-Impulsive Model (RIM) distinguishes between a reflective system, which is responsible for deliberate control, inhibition, and planning, and an impulsive system that drives automatic responses, often in conflict with reflective goals (Strack and Deutsch, 2004[176]). Similarly, the Dual Systems Model of Self-Control highlights the importance of EFs such as inhibition, working memory, and cognitive shifting in regulating impulses (Hofmann, Friese, and Strack, 2009[87]). Taken together, these models suggest that successful behavior change depends on the dynamic balance between fast, automatic impulses and slower, deliberate control processes, with successful regulation requiring both strong EFs and effective strategies for aligning impulsive tendencies with reflective goals.

Stages-based models conceptualize behavior change as a process that unfolds over time, with individuals progressing through distinct phases on the path from intention to action. The Health Action Process Approach (HAPA) emphasizes both action planning and coping planning, highlighting how individuals prepare for challenges and obstacles before engaging in behavior (Schwarzer and Luszczynska, 2008[164]). The Temporal Self-Regulation Model stresses the role of self-regulatory capacity, particularly inhibition, flexibility, and updating, in determining whether intentions are successfully translated into actions within temporal contexts (Hall and Fong, 2007[80]). Moreover, the Precaution Adoption Process Model focuses on decision-making stages and describes how individuals move from unawareness or indifference to deliberate decisions and the eventual adoption of protective behaviors (Weinstein, Sandman, and Blalock, 2008[195]). Together, these models emphasize that behavior change is not instantaneous but involves sequential stages, requiring both cognitive resources and adaptive planning strategies to successfully progress from intention to sustained action.

Integrative models combine multiple theoretical perspectives to explain behavior change and self-regulation as the result of interactions among cognitive, affective, and contextual processes. The COM-B model frames behavior as emerging from the interplay between capacity, opportunity, and motivation, emphasizing the roles of attention, decision-making, and action planning (Michie, van Stralen, and West, 2011[132]). The Multi-Process Action Control model extends this view by incorporating behavioral, affective, and cognitive regulation, highlighting the diversity of processes that guide action (Rhodes, 2021[155]). The Integrated Behavior Change Model, an extension of the Theory of Planned Behavior, places action planning at the core of intentional behavior change (Hagger and Chatzisarantis, 2014[79]). Similarly, the I-Change Model highlights the need for action planning alongside mechanisms to overcome barriers, as well as EFs such as inhibition, flexibility, and working memory (De Vries, 2017[44]). Finally, the Virtuous Circle Model emphasizes the cyclical role of EFs, including inhibition, updating, shifting, and attentional control, in sustaining self-regulation (or change) over time (Audiffren and André, 2019[7]). Collectively, these models converge on the idea that effective self-regulation requires not only deliberate planning but also the integration of executive capacities, emotional processes, and contextual opportunities, forming a dynamic and adaptive system of behavior regulation. 

The virtuous circle differs from other models in three ways: First, it is not limited to the intention-action transition but emphasizes the sustainability of behavior over time. Moreover, it is a circular model in which behavior itself becomes an important lever for change. Finally, it proposes an original hypothesis, the *effort hypothesis*, which considers effort as a useful investment rather than a cost that people systematically try to avoid.

Despite their theoretical diversity, all these models converge on the idea that EFs constitute a fundamental foundation for behavioral change. Together, they show that behavior change relies on a combination of EFs, emotional regulation, and strategies adapted to different goals. The next section details the mechanisms of action of the virtuous circle model.

## 8. Adherence to Exercise within the Virtuous Circle

Behavior change is a dynamic process that unfolds through successive, transient modifications. In the study of behavior change and health promotion, exercise adherence is a central concept, as it captures these evolving adjustments. Unlike compliance, which measures behavior in isolation, adherence reflects the reciprocal alignment of attitudes and behaviors. In other words, engaging in exercise can foster positive attitudinal adjustments, whereas favorable attitudes reinforce behavioral maintenance. The virtuous circle model conceptualizes adherence as the strength of the attitude-behavior link, positioning exercise not only as an outcome but also as a determinant of change (André et al., 2024[5]). From this perspective, adherence can be understood as the degree to which behaviors are consistent with attitude and vice versa. Individuals may initially exercise despite negative attitudes-perceiving the activity as tedious-but engagement can gradually foster supportive beliefs. Conversely, favorable attitudes toward physical activity, which some call “intention strength” (e.g., Conner and Norman, 2024[38]), facilitate repeated participation, capturing the dynamic processes underlying behavioral transformation.

Exercise can strengthen adherence through three interrelated mechanisms. This conceptualization has practical implications for rehabilitation, reconditioning, performance-based training, and preventive health programs. Rather than treating attitudes (or intention strength) solely as precursors, interventions can leverage exercise behavior itself as a driver of adherence. In this framework, exercise becomes an active determinant of change rather than a passive outcome.

### 8.1. Sustained effort

Long-term adherence requires sustained physical and cognitive effort. As discussed, chronic exercise improves cognitive control, which relies on EFs, self-control, and self-regulation to regulate thoughts, emotions, and actions (e.g., Audiffren et al., 2022[8]). Enhanced cognitive control in turn facilitates adherence by reducing the cognitive cost of effortful decisions and increasing the reward value of continued engagement. The virtuous circle model illustrates the bidirectional relationship between exercise and cognition: exercise strengthens executive functioning, which in turn improves self-regulation and reduces the appeal of sedentary alternatives. Repeated exercise also induces physiological and psychological adaptations-enhanced cardiovascular fitness, greater willpower, and increased valuation of effort-that reinforce engagement (e.g., Feil et al., 2021[62]). Effective strategies include implementation intentions (planning where, when, and how to exercise), self-monitoring (tracking steps, heart rate, or perceived exertion), goal setting and problem-solving to overcome barriers, and the use of contextual cues to build routines (e.g., Gollwitzer, 1999[75]; Sheeran et al., 2005[166]). These strategies transform exercise from a cognitively costly choice into habitual, semiautomatic behavior, strengthening long-term adherence. This repeated link enhances executive control processes, establishing a virtuous cycle in which improved cognitive control reduces the perceived cost of effort, further supporting adherence.

Recently, additional evidence has shown the effects of exercise on brain connectivity (Morris et al., 2022[138]; Ai et al., 2023[2]). These two studies were carried out with older adults and revealed that adherence to a healthy lifestyle is associated with specific brain characteristics. On the one hand, Morris et al. (2022[138]) demonstrated that higher nodal rs-FC within the DMN, CEN, and DAN predicted exercise adherence, whereas rs-FC between the ACC and the supplementary motor area (SMA), as well as between the right AI and the left temporoparietal /temporo-occipital junction, predicted changes in sedentary time in the walking group. On the other hand, Ai et al. (2023[2]) reported that compared with an adherent lifestyle group, a nonadherent lifestyle group exhibited greater positive correlations and fewer anticorrelations between the DMN and other task-positive networks (e.g., the DAN). Overall, a multimodal model combining structural, functional, and clinical measures achieved the strongest prediction, suggesting that adherence emerges from the interaction of executive control, self-reflection, and attentional regulation.

Sustained effort and adherence reinforce each other: exercise enhances executive control and self-regulation, which lowers the perceived cost of effort and make continued engagement more rewarding. Neurobiological evidence shows that adherence is supported by strengthened cortical and functional connectivity in networks linked to control, attention, and self-reflection, highlighting a virtuous cycle that promotes long-term lifestyle change.

### 8.2. Immediate motivation

High dropout rates in exercise interventions often result from the immediate costs of exercise (e.g., fatigue, discomfort, time, financial burden) outweighing delayed benefits (e.g., health improvements, weight loss). The initiation of exercise requires proximal motivators, such as increased arousal, pleasant bodily sensations (warmth, relaxation), enhanced mood, achievement of meaningful goals, and social interaction (Lee et al., 2016[111]). While these effects are short-term, they are crucial for initiating and reinforcing motivation. Behavior change techniques (BCTs), such as rewards, graded tasks, feedback, and biofeedback, enhance short-term motivation across populations, including cancer patients, individuals with obesity, and those experiencing cognitive decline (e.g., Carey et al., 2019[28]; Howlett et al., 2019[90]). Digital tools-wearable devices and AI-based applications-further support motivation by providing real-time feedback during sessions (e.g., Michaelsen and Esch, 2022[131]). By focusing on immediate reinforcers, practitioners can promote adherence at the level of each exercise session, laying the foundation for longer-term commitment.

### 8.3. Behavior-driven attitude change

Attitudes toward exercise often evolve more slowly than do behaviors. Relying solely on attitudinal change as a precursor may limit intervention effectiveness. Behavior itself can drive attitude change through mechanisms such as cognitive dissonance (Festinger, 1957[64]) and commitment theory (Kiesler, 1971[103]; Joule and Beauvois, 1998[101]). Cognitive dissonance arises when individuals act inconsistently with their beliefs; for example, a person who perceives exercise as unnecessary but nonetheless participates may later adjust their beliefs to justify the behavior. Commitment theory suggests that repeated, effortful engagement strengthens psychological investment and aligns attitudes with behavior. Effort is central to this process on the basis of two mechanisms: (a) effort justification (Aronson and Mills, 1959[6]), whereby individuals assign greater value to outcomes requiring substantial effort, legitimizing the cost invested; and (b) the effort heuristic, where people assume that effortful actions yield superior outcomes, enhancing perceived benefits and satisfaction. Consequently, exercise acts not only as a health intervention but also as a catalyst for attitudinal change, progressively reinforcing adherence.

Another way in which behavior can drive attitude change might be through the effect of exercise on changes in rs-FC within the DMN. For instance, self-efficacy belongs to self-beliefs and self-concepts that are associated with the rs-FC of several regions within the DMN (e.g., Davey et al., 2019[43]; Dixon and Gross, 2021[49]; Wang et al., 2022[192]). Self-efficacy refers to an individual's confidence in his or her ability to perform activities of a given intensity and duration, whether under optimal conditions or when facing obstacles and adversity. This confidence not only influences exercise-related choices and behaviors, but is also strongly linked to adherence to exercise routines, physical adaptability, and psychological well-being (Medrano-Ureña et al., 2020[128]). For instance, McAuley et al. (2011[125]) demonstrated that individuals who begin an exercise program with stronger executive functioning and greater use of self-regulatory strategies tend to develop stronger beliefs in their exercise abilities, which subsequently promotes higher adherence. The connectivity changes induced by chronic exercise may reflect an increase in positive self-representations and a decrease in negative self-representations. In the future, it would be interesting to test the hypothesis that regular exercise induces changes in functional connectivity within the DMN, which in turn underlies lasting changes in attitudes toward exercise.

These three pathways-immediate motivation, sustained effort, and behavior-driven attitude change-are deeply interconnected. Immediate reinforcers sustain participation in individual sessions; repeated sessions enhance executive functioning and self-regulation; and accumulated effort reshapes attitudes, reinforcing long-term adherence. Adherence should thus be understood as the evolving strength of the link between attitudes and behaviors. Recognizing exercise as both an outcome and a determinant deepens theoretical models of behavior change and informs interventions promoting sustainable engagement. Given its effortful nature, exercise induces attitudinal change via cognitive dissonance and effort justification. Repeated engagement under conditions of free choice, perceived personal cost, and consistent effort strengthen commitment, and align attitudes with behavior. By emphasizing behavior-oriented approaches, practitioners can catalyze a self-reinforcing cycle where repeated activity fortifies the attitude-behavior link, ensuring sustainable adherence.

With respect to the virtuous circle model, the relationship between exercise and EFs or effortful control has been studied more than the relationship between executive functioning and behavior and its maintenance (i.e., exercise adherence). Since 2019, several articles have strongly reviewed this link and provided more evidence (Boat and Cooper, 2019[16]; Gray-Burrows et al., 2019[76]; Strobach et al., 2020[177]; Frye and Shapiro, 2021[67]; Gürdere et al., 2023[77]). Three main relationships can be drawn from these reviews: (1) Cognitive control and EFs help individuals engage in healthy behavior and stop unhealthy behavior; (2) Cognitive control and EFs are more important in the adoption phase and may be less important in the maintenance phase; and (3) Cognitive control and EFs can help move intention into action.

Exercise adherence emerges from the dynamic interaction of immediate motivation, sustained effort, and behavior-driven attitude change. Immediate rewards, such as positive feelings and social interaction, encourage early engagement, while repeated effort strengthens self-regulation and gradually turns exercise into a habit (Gardner and Rebar, 2019[68]). Over time, these behaviors reshape attitudes through cognitive dissonance and commitment, creating a self-reinforcing cycle that consolidates long-term adherence.

## 9. Conclusions and Perspectives

The main objective of this article was to update the model of the virtuous circle linking physical exercise and cognition published in March 2019. The first version of the model considered only changes in functional connectivity induced by regular physical exercise between the SN and the CEN. The new version of the model now integrates the DMN, as it plays a fundamental role in the ability to remain engaged in an activity both in the present moment and over the long term.

This updated version of the model also specifies five neurobiological mechanisms that may synergistically contribute to maintaining the virtuous circle, whereas the previous version focuses mainly on the *neurotrophic hypothesis* and the *effort hypothesis*, with the latter remaining the mechanism that makes the greatest contribution to the virtuous circle. For instance, the *cardiovascular hypothesis*, which is grounded in the bidirectional interplay between the heart and the brain (i.e., the brain-heart axis), highlights the pivotal role of regular engagement in structured exercise, whether through high-intensity interval training, moderate aerobic activity, or resistance training, which exerts a regulatory effect on autonomic responses, enhances the production of BDNF, and fosters neurovascular coupling (e.g., Omole et al., 2025[140]). Collectively, these adaptations contribute to improved cognitive and cardiovascular resilience and facilitate the maintenance of exercise. Similarly, the *neurotrophic hypothesis* may be seen as reinforcing the *effort hypothesis*, insofar as neurotrophic factors released through exercise facilitate structural and functional connectivity changes within networks underlying cognitive control and positive self-representational beliefs.

Furthermore, two additional bidirectional relationships may contribute to this virtuous cycle: the relationship between exercise and sleep quality (e.g., Qian et al., 2025[152]), and the relationship between the brain and the gut (e.g., Cutuli et al., 2023[41]). On the one hand, exercise improves sleep quality (e.g., Zhou et al., 2025[204]); in turn, good sleep quality is associated with energy restoration and the consolidation of memory traces (e.g., Porkka-Heiskanen and Kalinchuk, 2011[150]; Weighall and Kellar, 2023[194]), thereby facilitating further engagement in exercise. On the other hand, exercise positively impacts the bidirectional communication between the brain and the gut, referred to as the gut-brain axis, which relies primarily on the direct and indirect interactions of the gut microbiota with the CNS, the autonomic nervous system, the enteric nervous system, and the HPA axis. This complex interplay involves multiple overlapping pathways, including neuroendocrine and immune mechanisms, and plays a pivotal role in neuronal development, brain function, cognitive regulation, and the aging process, subsequently participating in the maintenance of a virtuous circle between a behavior (i.e., exercising) and health.

The present article also reviews new experimental evidence supporting the relationship between the chronic effects of exercise and functional connectivity within and between the three major neural networks mentioned above. In the perspectives section presented in the previous article on the virtuous circle model, a lack of RCTs examining the effects of chronic exercise on the rs-FC of large-scale neural networks involved in cognitive control was mentioned. To date, only six new RCTs have been published; however, precise predictions regarding the networks positively impacted by chronic exercise have not been provided (see Supplementary Information, S2). Two articles targeted specific brain regions, the hippocampus (Broadhouse et al., 2020[23]) and the orbitofrontal cortex (Claus et al., 2023[34]), but neither refer to the three networks of the triple-network model in the rationale used to formulate their main hypothesis. However, three RCTs clearly supported the virtuous circle model (Voss et al., 2019[190]; Balazova et al., 2021[9]; Leocadi et al., 2024[112]; see section 6). In the future, researchers seeking to conduct RCTs to examine the chronic effects of exercise on rs-FC are encouraged to formulate very precise hypotheses concerning the networks involved, the type of connectivity (within-network or between-network), the direction of the effects (increased or decreased connectivity), and the theoretical rationale leading to these predictions.

Furthermore, the present article clarified the difference between the virtuous circle model and other behavior change models. In particular, the virtuous circle model proposes that attitudes and behavior (i.e., effort in exercise) are two key ways to initiate and stabilize health behavior (e.g., engaging in regular exercise). It emphasizes that the deployment of effort depends on the short-term benefits associated with regular physical activity; thus, people are willing to invest effort in physical activity as long as this effort is justified. This feature implies that any intervention involving physical activity must take these aspects into account to increase participants' adherence.

Over the past five years, several longitudinal studies with large samples have examined the bidirectional relationships between physical activity and cognitive and brain health, using different statistical approaches: cross-lagged panel models (Zhao et al., 2021[202]; Hofman et al., 2023[86]; Mitchell et al., 2025[133]), latent growth curve models (Stenling et al., 2022[173]), and mixed-model analyses (Maasakkers et al., 2021[120]). Three of these investigations confirmed reciprocal associations between exercise and cognition. For example, Zhao et al. (2021[203]) reported that physical performance predicted later cognitive outcomes, whereas cognition also predicted subsequent physical performance in 4,232 older Chinese adults. Similarly, Maasakkers et al. (2021[120]) reported in 1,276 Irish participants that each additional hour of sedentary behavior per day was linked to lower global cognition scores over time. Conversely, greater declines in immediate and delayed recall were related to slightly greater amounts of sedentary behavior and TV viewing after four years. By analyzing 2,888 adults from the United Kingdom, Mitchell et al. (2025[133]) reported that improvements in verbal memory increased the likelihood of being classified as mid-active during the next wave, whereas becoming physically active was associated with a modest improvement in subsequent memory scores. This reciprocal association appeared stronger for men. In contrast, the two remaining studies supported only a unidirectional effect. Stenling et al. (2022[173]) reported that higher baseline physical activity was associated with a lower decline in episodic memory among 1,722 Swedish adults, whereas in 4,365 Dutch participants, Hofman et al. (2023[86]) reported that greater brain volume at baseline predicted higher levels of physical activity at follow-up. Taken together, this emerging longitudinal evidence using structural equation modeling provides promising insights into the dynamic links between exercise and cognition; however, further development is warranted.

The virtuous circle described herein contributes to the development of healthy habits that, in turn, facilitate its maintenance (e.g., Feil et al., 2021[62]). The mechanisms of action outlined above (i.e., sustained effort, immediate motivation, and changes in attitudes) promote the formation of habits. The earlier in the lifespan the virtuous cycle is initiated, the more solidly health-promoting habits that facilitate long-term maintenance are established. The virtuous circle that maintains engagement in regular physical exercise slows physical and cognitive aging. In contrast, a sedentary lifestyle fosters a vicious circle that tends to accelerate aging. Considering the theoretical framework of the virtuous circle model and *effort hypothesis* may, on the one hand, support the development of intervention programs aimed at rehabilitation and reconditioning-particularly for patients suffering from chronic fatigue, anhedonia, or apathy. Specifically, the implementation of exercise programs should be systematically combined with therapeutic patient education to strengthen the links between exercise behavior and a positive attitude toward physical activity. Beyond rehabilitation, the virtuous circle may also guide motivational interventions across various clinical contexts. When coupled with AI-assisted monitoring, they could enhance adherence, optimize feedback, and sustain the long-term benefits of physical activity.

## Declaration

### Conflict of interest

The authors declare that they have no conflicts of interest related to this work.

### Using Artificial Intelligence (AI)

AI (ChatGPT) was used for the conception of Figure 3(Fig. 3) and translation of sentences from French to English.

### Authors contribution

Conceptualization = MA, NA; Writing - Original Draft = MA, NA; Writing - Review and Editing = MA, NA; Visualization = MA; Funding acquisition = MA.

## Supplementary Material

Supplementary information

## Figures and Tables

**Table 1:**
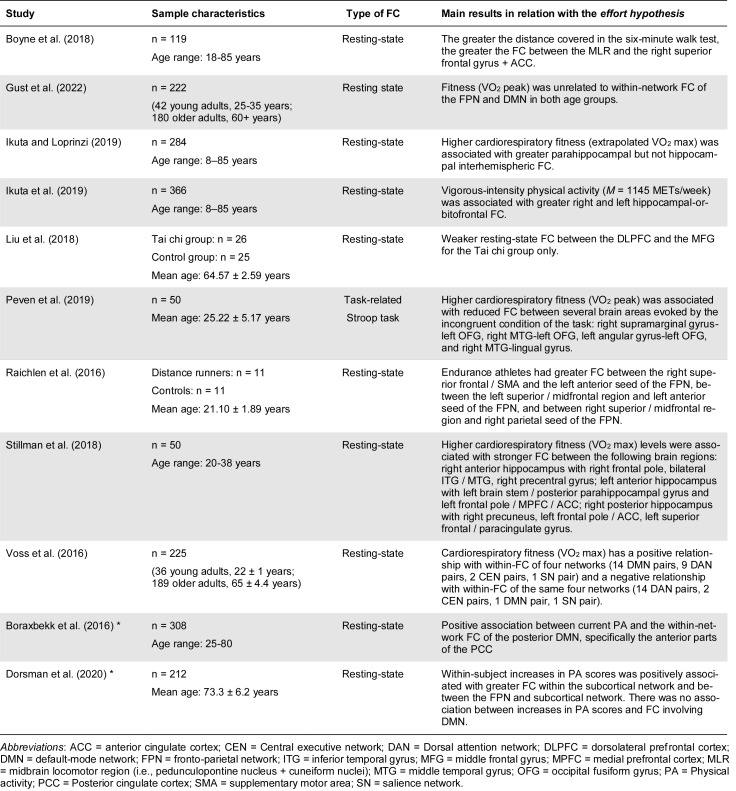
Main results of the cross-sectional studies examining the association between physical activity or physical fitness levels and functional connectivity within or between brain regions belonging to large-scale neuronal networks. * Longitudinal study

**Table 2:**
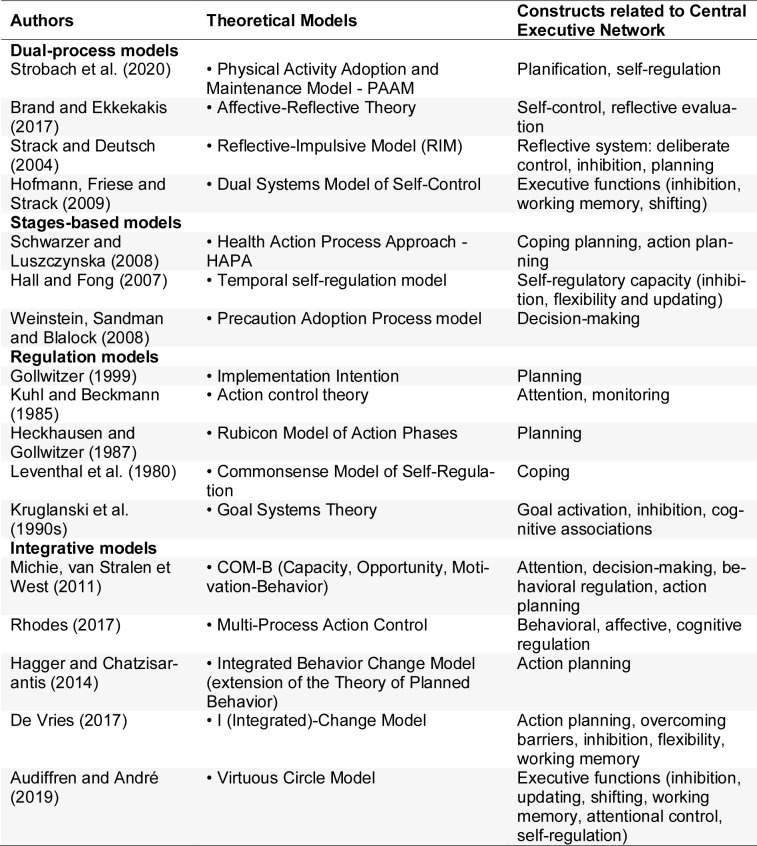
Executive functions that play an important role in the models of behavior change.
