# Supplementary material for: The effort hypothesis at the heart of the virtuous circle
Source: EXCLI J. 2025 Nov 12;24:1524–54. doi: 10.17179/excli2025-8937 (PMC12627994; doi:10.17179/excli2025-8937)
Supplement: Supplementary information [file EXCLI-24-1524-s-001.pdf]

EXCLI Journal 2025;24:1524-1554 – ISSN 1611-2156
Received: September 18, 2025, accepted: October 04, 2025, published: November 12, 2025

Supplementary information to:

Review article:

THE EFFORT HYPOTHESIS AT THE HEART OF THE VIRTUOUS CIRCLE

Michel Audiffren*, Nathalie André

Centre de Recherches sur la Cognition et l'Apprentissage, Université de Poitiers, CNRS, Poitiers, France

* **Corresponding author:** Michel Audiffren, UMR 7295 CeRCA, Centre de Recherches sur la Cognition et l'Apprentissage, CNRS – Université de Poitiers, Bât A5 – TSA 21103, 5 rue Théodore Lefebvre, 86073 Poitiers Cedex, France.
E-mail: michel.audiffren@univ-poitiers.fr

https://dx.doi.org/10.17179/excli2025-8937

This is an Open Access article distributed under the terms of the Creative Commons Attribution License (https://creativecommons.org/licenses/by/4.0/).

S1: Intervention studies examining the effect of chronic exercise on rs-FC within and between large-scale neuronal networks

List of the thirty-seven intervention studies examining the effect of chronic exercise on resting-state functional connectivity:

1*. Balazova Z, Marecek R, Novakova L, Nemcova-Elfmarkova N, Kropacova S, Brabenec L, et al. Dance intervention impact on brain plasticity: A randomized 6-month fMRI study in non-expert older adults. Front Aging Neurosci. 2021;13:724064. doi: 10.3389/fnagi.2021.724064.

2. Baniqued PL, Gallen CL, Voss MW, Burzynska AZ, Wong CN, Cooke GE, et al. Brain network modularity predicts exercise-related executive function gains in older adults. Front Aging Neurosci. 2018;9:426. doi: 10.3389/fnagi.2017.00426.

3*. Broadhouse KM, Singh MF, Suo C, Gates N, Wen W, Brodaty H, et al. Hippocampal plasticity underpins long-term cognitive gains from resistance exercise in MCI. Neuroimage Clin. 2020;25:102182. doi: 10.1016/j.nicl.2020.102182.

4*. Burdette JH, Laurienti PJ, Espeland MA, Morgan A, Telesford Q, Vechlekar CD, et al. Using network science to evaluate exercise-associated brain changes in older adults. Front Aging Neurosci. 2010;2:23. doi: 10.3389/fnagi.2010.00023.

5. Chirles TJ, Reiter K, Weiss LR, Alfini AJ, Nielson KA, Smith JC. Exercise training and functional connectivity changes in mild cognitive impairment and healthy elders. J Alzheimers Dis. 2017;57(3):845-856. doi: 10.3233/JAD-161151.

6*. Claus J, Upadhyay N, Maurer A, Klein J, Scheef L, Daamen M, et al. Physical activity alters functional connectivity of orbitofrontal cortex subdivisions in healthy young adults: A longitudinal fMRI study. Healthcare (Basel). 2023;11(5):689. doi: 10.3390/healthcare11050689.

7. Demirakca T, Cardinale V, Dehn S, Ruf M, Ende G. The exercising brain: Changes in functional connectivity induced by an integrated multimodal cognitive and whole-body coordination training. Neural Plast. 2016;2016:8240894. doi: 10.1155/2016/8240894.

8*. Dimitriadis SI, Castells-Sánchez A, Roig-Coll F, Dacosta-Aguayo R, Lamonja-Vicente N, Torán-Monserrat P, et al. Intrinsic functional brain connectivity changes following aerobic exercise, computerized cognitive training, and their combination in physically inactive healthy late-middle-aged adults: the Projecte Moviment. Geroscience. 2024;46(1):573-596. doi: 10.1007/s11357-023-00946-8.

9.* Eyre HA, Acevedo B, Yang H, Siddarth P, Van Dyk K, Ercoli L, et al. Changes in neural connectivity and memory following a Yoga intervention for older adults: A pilot study. J Alzheimers Dis. 2016;52(2):673-84. doi: 10.3233/JAD-150653.

10*. Flodin P, Jonasson LS, Riklund K, Nyberg L, Boraxbekk CJ. Does aerobic exercise influence intrinsic brain activity? An aerobic exercise intervention among healthy old adults. Front Aging Neurosci. 2017;9:267. doi: 10.3389/fnagi.2017.00267.

11. Gui W, Cui X, Miao J, Zhu X, Li J. The effects of simultaneous aerobic exercise and video game training on executive functions and brain connectivity in older adults. Am J Geriatr Psychiatry. 2024;32(10):1244-1258. doi: 10.1016/j.jagp.2024.04.009.

12*. Hsu CL, Best JR, Wang S, Voss MW, Hsiung RGY, Munkacsy M, et al. The impact of aerobic exercise on fronto-parietal network connectivity and its relation to mobility: An exploratory analysis of a 6-month randomized controlled trial. Front Hum Neurosci. 2017;11:344. doi: 10.3389/fnhum.2017.00344. Erratum in: Front Hum Neurosci. 2017;11:449. doi: 10.3389/fnhum.2017.00449.

13. Iosipchuk O, Wylie GR, Motl RW, Sandroff BM. Aerobic exercise training and depressive symptoms in people with multiple sclerosis: Brief report on default-mode network resting-state functional connectivity. Int J MS Care. 2025;27(Q1):34-41. doi: 10.7224/1537-2073.2024-003.

14. Ji L, Zhang H, Potter GG, Zang YF, Steffens DC, Guo H, et al. Multiple neuroimaging measures for examining exercise-induced neuroplasticity in older adults: A quasi-experimental study. Front Aging Neurosci. 2017;9:102. doi: 10.3389/fnagi.2017.00102.

15. Johansson ME, Cameron IGM, Van der Kolk NM, de Vries NM, Klimars E, Toni I, et al. Aerobic exercise alters brain function and structure in Parkinson’s disease: A randomized controlled Trial. Ann Neurol. 2022;91(2):203-216. doi: 10.1002/ana.26291.

16. Legget KT, Wylie KP, Cornier MA, Melanson EL, Paschall CJ, Tregellas JR. Exercise-related changes in between-network connectivity in overweight/obese adults. Physiol Behav. 2016;158:60-7. doi: 10.1016/j.physbeh.2016.02.031.

17*. Leocadi M, Canu E, Sarasso E, Gardoni A, Basaia S, Calderaro D, et al. Dual-task gait training improves cognition and resting-state functional connectivity in Parkinson's disease with postural instability and gait disorders. J Neurol. 2024;271(4):2031-2041. doi: 10.1007/s00415-023-12151-w.

18. Li R, Zhu X, Yin S, Niu Y, Zheng Z, Huang X, et al. Multimodal intervention in older adults improves resting-state functional connectivity between the medial prefrontal cortex and medial temporal lobe. Front Aging Neurosci. 2014;6:39. doi: 10.3389/fnagi.2014.00039.

19. Li Y, Wu K, Hu X, Xu T, Li Z, Zhang Y, et al. Altered effective connectivity of resting-state networks by Tai Chi Chuan in chronic fatigue syndrome patients: A multivariate Granger causality study. Front Neurol. 2022;13:858833. doi: 10.3389/fneur.2022.858833.

20. Liu J, Tao J, Xia R, Li M, Huang M, Li S, et al. Mind-body exercise modulates locus coeruleus and ventral tegmental area functional connectivity in individuals with mild cognitive impairment. Front Aging Neurosci. 2021;13:646807. doi: 10.3389/fnagi.2021.646807.

21. Lloyd KM, Morris TP, Anteraper S, Voss M, Nieto-Castanon A, Whitfield-Gabrieli S, et al. Data-driven MRI analysis reveals fitness-related functional change in default mode network and cognition following an exercise intervention. Psychophysiology. 2024;61(4):e14469. doi: 10.1111/psyp.14469.

22*. Magon S, Donath L, Gaetano L, Thoeni A, Radue EW, Faude O, et al. Striatal functional connectivity changes following specific balance training in elderly people: MRI results of a randomized controlled pilot study. Gait Posture. 2016;49:334-339. doi: 10.1016/j.gaitpost.2016.07.016.

23. McGregor KM, Crosson B, Krishnamurthy LC, Krishnamurthy V, Hortman K, Gopinath K, et al. Effects of a 12-week aerobic spin intervention on resting state networks in previously sedentary older adults. Front Psychol. 2018;9:2376. doi: 10.3389/fpsyg.2018.02376.

24*. Pieramico V, Esposito R, Sensi F, Cilli F, Mantini D, Mattei PA, et al. Combination training in aging individuals modifies functional connectivity and cognition, and is potentially affected by dopamine-related genes. PLoS One. 2012;7(8):e43901. doi: 10.1371/journal.pone.0043901.

25*. Prehn K, Lesemann A, Krey G, Witte AV, Köbe T, Grittner U, et al. Using resting-state fMRI to assess the effect of aerobic exercise on functional connectivity of the DLPFC in older overweight adults. Brain Cogn. 2019;131:34-44. doi: 10.1016/j.bandc.2017.08.006.

26. Stellmann JP, Maarouf A, Schulz KH, Baquet L, Pöttgen J, Patra S, et al. Aerobic exercise induces functional and structural reorganization of CNS networks in multiple sclerosis: A randomized controlled trial. Front Hum Neurosci. 2020;14:255. doi: 10.3389/fnhum.2020.00255.

27*. Suo C, Singh MF, Gates N, Wen W, Sachdev P, Brodaty H, et al. Therapeutically relevant structural and functional mechanisms triggered by physical and cognitive exercise. Mol Psychiatry. 2016;21(11):1633-1642. doi: 10.1038/mp.2016.19.

28*. Tao J, Liu J, Egorova N, Chen X, Sun S, Xue X, et al. Increased hippocampus–medial prefrontal cortex resting-state functional connectivity and memory function after Tai Chi Chuan practice in elder adults. Front. Aging Neurosci. 2016;8:25. doi: 10.3389/fnagi.2016.00025.

29*. Tao J, Chen X, Egorova N, Liu J, Xue X, Wang Q, et al. Tai Chi Chuan and Baduanjin practice modulates functional connectivity of the cognitive control network in older adults. Sci Rep. 2017;7:41581. doi: 10.1038/srep41581.

30*. Tozzi L, Carballedo A, Lavelle G, Doolin K, Doyle M, Amico F, et al. Longitudinal functional connectivity changes correlate with mood improvement after regular exercise in a dose-dependent fashion. Eur J Neurosci. 2016;43(8):1089-96. doi: 10.1111/ejn.13222.

31*. Voss MW, Prakash RS, Erickson KI, Basak C, Chaddock L, Kim JS, et al. Plasticity of brain networks in a randomized intervention trial of exercise training in older adults. Front Aging Neurosci. 2010;2:32. doi: 10.3389/fnagi.2010.00032.

32*. Voss MW, Sutterer M, Weng TB, Burzynska AZ, Fanning J, Salerno E, et al. Nutritional supplementation boosts aerobic exercise effects on functional brain systems. J Appl Physiol (1985). 2019;126(1):77-87. doi: 10.1152/japplphysiol.00917.2017.

33. Voss MW, Weng TB, Narayana-Kumanan K, Cole RC, Wharff C, Reist L, et al. Acute exercise effects predict training change in cognition and connectivity. Med Sci Sports Exerc. 2020;52(1):131-140. doi: 10.1249/MSS.0000000000002115.

34. Won J, Callow DD, Pena GS, Jordan LS, Arnold-Nedimala NA, Nielson KA, et al. Hippocampal functional connectivity and memory performance after exercise intervention in older adults with mild cognitive impairment. J Alzheimers Dis. 2021a;82(3):1015-1031. doi: 10.3233/JAD-210051.

35. Won J, Faroqi-Shah Y, Callow DD, Williams A, Awoyemi A, Nielson KA, et al. Association between greater cerebellar network connectivity and improved phonemic fluency performance after exercise training in older adults. Cerebellum. 2021b;20(4):542-555. doi: 10.1007/s12311-020-01218-3.

36. Wu K, Li Y, Zou Y, Ren Y, Wang Y, Hu X, et al. Tai Chi increases functional connectivity and decreases chronic fatigue syndrome: A pilot intervention study with machine learning and fMRI analysis. PLoS One. 2022;17(12):e0278415. doi: 10.1371/journal.pone.0278415.

37*. Zhu L, Xiong X, Dong X, Zhao Y, Kawczyński A, Chen A, et al. Working memory network plasticity after exercise intervention detected by task and resting-state functional MRI. J Sports Sci. 2021;39(14):1621-1632. doi: 10.1080/02640414.2021.

* Article included in the narrative review.

EXCLI Journal 2025;24:1524-1554 – ISSN 1611-2156
Received: September 18, 2025, accepted: October 04, 2025, published: November 12, 2025

S1: Intervention studies examining the effect of chronic exercise on rs-FC within and between large-scale neuronal networks

#	Reference	Type of study	Type of FC	Intervention	Sample characteristics	Main results
**1**	Balazova et al. (2021) *	RCT	Resting-state	Design: Exercise vs control<br>Exercise: Dance<br>Control: Life as usual<br>Program duration: 6 months<br>Intensity: Moderate<br>Session duration: 60 min<br>Session frequency: 3 times a week	Exercise: n = 36<br>Control: n= 32<br>Mean age: 69.11 ± 5.76 years	The within-network rs-FC in the precuneus (DMN) increased in the dance group and decreased in the control group. The between-network rs-FC increased between SN and CEN in the dance group and decreased in the control group. Positive correlation between changes in rs-FC between SN and CEN and changes in executive functions.
**2**	Baniqued et al. (2018)	RCT	Resting-state	Design: Four arms<br>1. Aerobic exercise (AE)<br>2. Aerobic exercise + nutritional supplement (AEN)<br>3. Stretching, strengthening and stability (SSS)<br>4. Dance<br>Program duration: 6 months<br>Intensity (AE): from 60% to 60-75% MHR<br>Session duration: 60 min<br>Session frequency: 3 times a week	AE: n = 29<br>AEN: n = 29<br>SSS: n = 32<br>Dance: n = 38<br>Mean age: 64.74 ± 4.11 years	Training effects not analyzed.
**3**	Broadhouse et al. (2020) *	RCT	Resting-state	Design: Four arms<br>1. Progressive resistance and computerized cognitive training (PRT + CCT)<br>2. PRT + sham CCT<br>3. CCT + sham PRT<br>4. Sham PRT + sham CCT (SHAM + SHAM)<br>Program duration: 26 weeks<br>Follow-up: 12 months	PRT + CCT: n = 20<br>PRT + sham CCT: n = 13<br>CCT + sham PRT: n = 19<br>SHAM + SHAM: n = 22<br>Mean age: 69.5 ± 6.6 years	Rs-FC between left PCC and hippocampus (i.e., within-network DMN connectivity) was significantly strengthened in PRT + CCT and PRT + SHAM compared to CCT + SHAM and SHAM + SHAM over the 18-month period.

EXCLI Journal 2025;24:1524-1554 – ISSN 1611-2156
Received: September 18, 2025, accepted: October 04, 2025, published: November 12, 2025

				Intensity (PRT): High intensity<br>Session duration: 90 min<br>Session frequency: 2-3 times a week		
**4**	Burdette et al. (2010) *	RCT	Resting-state	Design: Exercise vs Control<br>Exercise: Aerobic<br>Control: Health education-based lectures + light stretching<br>Program duration: 4 months<br>Intensity: 12-14 RPE<br>Session duration: 40 min<br>Session frequency: 2 times a week	Exercise: 6<br>Control: 5<br>Mean age: 75.96 ± 4.08 years	Increased between-network rs-FC between the hippocampus (DMN) and the ACC (SN) in the exercise group that was not observed in the control group.
**5**	Chirles et al. (2017)	UT	Resting state	Design: Exercise alone<br>Exercise: Walking<br>Program duration: 12 weeks<br>Intensity: 50-60% of HRR<br>Session duration: 30 min<br>Session frequency: 4 times a week	MCI: n = 16<br>HC: n = 16<br>Mean age: 77.85 ± 7.00 years	Increased between-network FC between PCC/precuneus (DMN) and four brain regions of the CEN in MCI group only: right middle frontal gyrus, right superior frontal gyrus, left IPL, right IPL.
**6**	Claus et al. (2023) *	RCT	Resting-state	Design: Exercise vs control<br>Exercise: Interval running<br>Control: Maintaining current lifestyle<br>Program duration: 6 months<br>Intensity: 75-80% MHR<br>Session duration: 25-45 min<br>Session frequency: 3 times a week	Exercise = n = 18<br>Control: n = 10<br>Mean age: 23.83 ± 4.01 years	Decreased between-network FC between OFC (extended DMN) and the left DLPFC (CEN) in the exercise group and the reverse in the Control group after 6 months. Increased between-network FC between OFC (extended DMN) and the right MFG (CEN) in the exercise group after 6 months. No effect in the Control group.
**7**	Demirakca et al. (2016)	QES	Resting-state	Design: Exercise vs Control<br>Exercise: Exergames (Life kinetik)<br>Control: Life as usual<br>Program duration: 13 weeks	Exercise: n = 21<br>Control: n = 11<br>Mean age: 48.34 ± 8.68 years	Increased within-network FC within the CEN in the exercise group but not in the Control group after 13 weeks: Right DLPFC (BA9) – right supramarginal gyrus (BA40); right

				Intensity:<br>Session duration: 60 min<br>Session frequency: 1 time a week		MCG - right anterior prefrontal cortex (BA10) & DLPFC (BA9).<br>Increased between-network FC between the left FEF / SFG (DAN) and the ventral ACC (extended DMN) in the exercise group but not in the Control group after 13 weeks.
**8**	Dimitriadis et al. (2024) *	RCT	Resting-state	Design: Four arms<br>1. Aerobic exercise (AE), brisk walking<br>2. Computerized cognitive training (CCT)<br>3. Combined training (COMB)<br>4. Life as usual<br>Program duration: 12 weeks<br>Intensity: 9-10 RPE<br>Session duration: 30-45 min<br>Session frequency: 5 times a week	AE: n = 25<br>CCT: n = 23<br>COMB: n = 19<br>Control: n = 15<br>Mean age: 58.38 ± 5.47 years	Only the results of the AE group are presented. Increased rs-FC between the right STG (VAN) and left MTG (posterior DMN), and between the left and right inferior frontal gyri (orbital part) with left frontal middle gyrus (orbital part) (anterior DMN). Decreased rs-FC between the left fusiform gyrus (posterior DMN) and right middle temporal gyrus (posterior DMN), between the left hippocampus (DMN) and left and right precentral gyrus (posterior DMN), and between the left and right SMAs and the right thalamus.
**9**	Eyre et al. (2016) *	RCT	Resting-state	Design: Two arms<br>1: Yoga<br>2: Memory enhancement training (MET)<br>Program duration: 12 weeks<br>Intensity: NS<br>Session duration: 60 min<br>Session frequency: 1 time a week	Yoga: n = 14<br>MET: n = 11<br>Mean age: 67.41 ± 9.59 years	Increased rs-FC within the DMN correlated with improved verbal memory performance.
**10**	Flodin et al. (2017) *	RCT	Resting-state	Design: Exercise vs Control<br>Exercise: Aerobic exercise<br>Control: Stretching and Toning<br>Program duration: 6 months	Exercise: n = 22<br>Control: n = 25<br>Mean age: 68.81 ± 2.82 years	Gains in aerobic capacity due to training predicted increased rs-FC between the right medial temporal lobe (DMN) and frontal and parietal regions (CEN).

EXCLI Journal 2025;24:1524-1554 – ISSN 1611-2156
Received: September 18, 2025, accepted: October 04, 2025, published: November 12, 2025

				Intensity: from 40 to 80% MHR<br>Session duration: 30-60 min<br>Session frequency: 3 times a week		
**11**	Gui et al. (2024)	QES	Resting-state	Design: Four arms<br>1. Cognitive training (CT)<br>2. Aerobic exercise (AE)<br>3. Combination of aerobic exercise and videogame training (CCA)<br>4. Passive control<br>Program duration: 4 months<br>Intensity: 50-70% MHR (AE and CCA)<br>Session duration: 60 min<br>Session frequency: 2 times a week	CT: 23<br>AE: 22<br>CCA: 21<br>Control: 21<br>Mean age: 62.90 ± 4.58 years	No effect of the intervention on rs-FC but significant correlation between change in EF and change in within-network rs-FC between the left and right CEN.
**12**	Hsu et al. (2017) *	RCT	Task-related Finger tap-ping task	Design: Exercise vs Control<br>Exercise: Aerobic, walking<br>Control: Usual care<br>Program duration: 6 months<br>Intensity: 60-70% HRR<br>Session duration: 60 min<br>Session frequency: 3 times a week	Exercise: n = 12<br>Control: n = 9<br>Mean age: 71.10 ± 8.86 years	Reduced within-network rs-FC within the DAN was associated with greater cardiovascular capacity and im-proved mobility performance.
**13**	Iosipchuk et al. (2025)	RCT	Resting-state	Design: Exercise vs Control<br>Exercise: Aerobic, treadmill walking<br>Control: Stretching and range-of-motion<br>Program duration: 12 weeks<br>Intensity: NS<br>Session duration: NS<br>Session frequency: 3 times a week	Exercise: 5<br>Control: 5<br>Mean age: 46.60 ± 12.12 years	Training effects not reported.

EXCLI Journal 2025;24:1524-1554 – ISSN 1611-2156
Received: September 18, 2025, accepted: October 04, 2025, published: November 12, 2025

**14**	Ji et al. (2017)	QES	Resting-state	Design: Exercise vs Control<br>Exercise: Exergames, Wii-fit<br>Control: Life as usual, waiting list<br>Program duration: 6 weeks<br>Intensity: NS<br>Session duration: 30 min<br>Session frequency: 5 times a week	Exercise: 12<br>Control: 12<br>Mean age: 70 ± 7.78 years	Changes in rs-FC induced by exergames but not between SN, CEN and DMN.
**15**	Johansson et al. (2022)	RCT	Resting-state	Design: Exercise vs Control<br>Exercise: Aerobic, cycling (AE)<br>Control: Stretching, flexibility, relaxation<br>Program duration: 6 months<br>Intensity: 50-80% HRR (AE)<br>Session duration: 30-45 min<br>Session frequency: 3 times a week	Exercise: n = 21<br>Control: n = 25<br>Mean age: 59.39 ± 9.57 years	Examination of the rs-FC between striatal subregions (putamen, caudate nucleus, and nucleus, accumbens) and the sensorimotor network.
**16**	Legget et al. (2016)	UT	Resting-state	Design: Exercise alone<br>Exercise: Aerobic, supervised treadmill-walking<br>Program duration: 6 months<br>Intensity: 60% to 75%<br>Session duration: from ~15–20 min/day to 40–60 min/day<br>Session frequency: 5 times a week	Exercise: n = 11 overweight / obese individuals<br>Mean age: 38.2 ± 3.2 years	Reductions in outgoing causal flow from PCC to left CEN and posterior DMN following exercise. Changes in connectivity between PCC (posterior DMN) and the ventral were significantly correlated with change in VO_2max.
**17**	Leocadi et al. (2024) *	RCT	Resting-state	Design: 2 arms<br>1. Dual-task gait/balance with action observation training (AOT) and motor imagery<br>(MI)<br>2. Dual-task gait/balance only	Parkinson's disease patients with postural instability and gait disorders.<br>Dual-task + AOT + MI: n = 11<br>Dual-task: n = 10<br>Mean age: 66.09 ± 8.09 years	Dual-task + AOT-MI group showed increased rs-FC of the left anterior prefrontal cortex within the anterior SN and reduced rs-FC of the right anterior prefrontal cortex within the anterior DMN compared to Dual-task group after exercise. In the Dual-task + AOT-MI group, reduced rs-FC of

EXCLI Journal 2025;24:1524-1554 – ISSN 1611-2156
Received: September 18, 2025, accepted: October 04, 2025, published: November 12, 2025

				Program duration: 6 weeks<br>Intensity: Light<br>Session duration: 60 min<br>Session frequency: 3 times a week		the right anterior prefrontal cortex within the anterior DMN was correlated with improved accuracy in the Attention Switching Task set-shifting condition after training.
**18**	Li et al. (2014)	QES	Resting-state	Design: Intervention + Control<br>Intervention: Cognitive training + Tai Chi Chuan + group counseling (CTC)<br>Control: Lectures<br>Program duration: 6 weeks<br>Intensity: Light<br>Session duration: 60 min<br>Session frequency: 3 times a week	CTC: n = 17<br>Control: n = 17<br>Mean age: 70.15 ± 4.92 years	The intervention group showed an increased rs-FC between the MPFC (DMN) and left parahippocampal cortex (DMN) after the training activities while in the control group, there was no significant change. This change in connectivity positively correlated with the change in performance in the Category Fluency Test.
**19**	Li et al. (2022)	UT	Resting-state	Design: Exercise only<br>Exercise: Tai Chi Chuan<br>Program duration: 4 weeks<br>Intensity: Light<br>Session duration: 60 min<br>Session frequency: 2 times a week	CFS: n = 21<br>HC: n = 19<br>Mean age: 35.49 ± 12.29 years	Increased rs-FC within the PCC (DMN) after training.
**20**	Liu et al. (2021)	RCT	Resting-state	Design: 3 arms<br>1. Baduanjin (BDJ)<br>2. Aerobic exercise (AE), walking<br>3. Control: Health education<br>Program duration: 24 weeks<br>Intensity BDJ: Light<br>Intensity AE: 55-75% HRR<br>Session duration: 60 min<br>Session frequency: 3 times a week	Baduanjin: n = 20<br>Walking: n = 17<br>Control: n = 20<br>Mean age: 65.61 ± 4.40 years	Increased rs-FC between right LC – right ACC (SN) in the Baduanjin group compared to the control and brisk walking groups. Increased rs-FC between the left and right LC and the right insula (SN) in the Baduanjin group compared to the control group. Increased rs-FC between left VTA and bilateral anterior insula (SN) in the Baduanjin group compared to the control group.
**21**	Lloyd et al. (2024)	RCT	Resting-state	Type: Three arms<br>1. Walking (AE)	Walking: n = 28<br>WNS: n = 31<br>Dancing: n = 40	Increased correlations between the precentral gyrus (SMN) and a series of clusters in the DAN (superior

EXCLI Journal 2025;24:1524-1554 – ISSN 1611-2156
Received: September 18, 2025, accepted: October 04, 2025, published: November 12, 2025

				2. Walking + nutritional supplement (WNS)<br>3. Dancing<br>4. Stretching and toning group<br>Program duration: 6 months<br>Intensity (AE and WNS): from 50%-60% to 60%-75% MHR<br>Session duration: 60 min<br>Session frequency: 3 times a week	Control: n = 40<br>Mean age: 65.04 ± 4.2 years	division of the bilateral LOC; right SPL) associated with pre-intervention cardiorespiratory fitness. Increased anti-correlations between the precentral gyrus (SMN) and a series of clusters in the DMN (bilateral IFG, right hippocampus, anterior division right STG) associated with pre-intervention cardiorespiratory fitness.
**22**	Magon et al. (2016) *	RCT	Resting-state	Design: Exercise vs Control<br>Exercise: Balance<br>Control: Health education<br>Program duration: 6 weeks<br>Intensity: Light<br>Session duration: 25-30 min<br>Session frequency: 3 times a week	Balance: n = 14<br>Control: n = 14<br>Mean age: 62.05 ± 5.35 years	No rs-FC differences before or after the training between the balance and Control groups.
**23**	McGregor et al. (2018)	RCT	Resting-state	Design: Exercise vs Control<br>Exercise: Aerobic<br>Control: Balance training<br>Program duration: 12 weeks<br>Intensity: 50-75% HRR<br>Session duration: 20-45 min<br>Session frequency: 3 times a week	Exercise: n = 19<br>Control: n = 18<br>Mean age: 73.5 ± 6.8 years	Increased rs-FC between the left primary motor cortex (SMN) to anterior DMN in the exercise group compared to balance group after 12 weeks of training.
**24**	Pieramico et al. (2012) *	RCT	Resting-state	Design: Intervention + Control<br>Intervention: Cognitive + aerobic + sensorial stimuli + fun recreational activities<br>Control: Life as usual<br>Program duration: 6 months<br>Intensity: NS	Intervention: n = 15<br>Control: n = 15<br>Age range: 60-75 years	Intervention increased rs-FC within the PCC (DMN) and FEF (DAN) and decreased rs-FC within precuneus (DMN) and right angular gyrus (DMN). Significant positive correlations were found between strength of rs-FC in PCC and global prose memory, immediate recall, delayed

EXCLI Journal 2025;24:1524-1554 – ISSN 1611-2156
Received: September 18, 2025, accepted: October 04, 2025, published: November 12, 2025

				Session duration: 60-120 min<br>Session frequency: 6 times a week		recall scores, and between FEF and immediate recall scores.
**25**	Prehn et al. (2019) *	RCT	Resting-state	Design: Exercise vs Control<br>Type: Aerobic, cycling<br>Control: Stretching and toning<br>Program duration: 6 months<br>Intensity: 80% AT<br>Session duration: 45 min<br>Session frequency: 2 times a week	Exercise: n = 11<br>Control: n = 18<br>Mean age: 66.52 ± 5.65 years	Increased rs-FC after training between left DLPFC (CEN) and SPG/precuneus (DMN) in the exercise group compared to the control group. Increased rs-FC after training between right DLPFC (CEN) and precuneus (DMN) in the exercise group compared to the control group.
**26**	Stellmann et al. (2020)	RCT	Resting-state	Design: Exercise vs Control<br>Exercise: Aerobic, cycling, interval training<br>Control: Waiting list<br>Program duration: 12 weeks<br>Intensity: High<br>Session duration: 20-70 min<br>Session frequency: 2-3 times a week	Multiple sclerosis patients<br>Exercise: n = 30<br>Control: n = 27<br>Mean age: 39.11 ± 9.96 years	Hub independent increased functional connectivity in the exercise group while the control group presented a loss of functional hub connectivity. No reporting of brain regions.
**27**	Suo et al. (2016) *	RCT	Resting-state	Type: Four arms<br>1. Progressive resistance training + computerized cognitive training (PRT + CCT)<br>2. PRT + Sham<br>3. CCT + Sham<br>4. Sham + Sham<br>Program duration: 26 weeks<br>Intensity: 15-18 RPE<br>Session duration: 90 min<br>Session frequency: 2 times a week	PRT + CCT: n = 21<br>PRT + Sham: n = 16<br>CCT + Sham: n = 20<br>Sham + Sham: n = 22<br>Mean age: 70.1 ± 6.7 years	PRT training led to decreased rs-FC between PCC (DMN) and ACC (SN) compared with non-PRT training. CCT training led to increased rs-FC between hippocampus (DMN) and left SFT (CEN) compared with non-CCT training. Increased rs-FC hippocampus–left SFT was positively correlated with improved overall memory performance.
**28**	Tao et al. (2016) *	RCT	Resting-state	Type: Three arms<br>1. Tai Chi Chuan<br>2. Baduanjin	Tai Chi Chuan: n = 21<br>Baduanjin: n = 16<br>Control: n = 25	After the 12-week practice, increased rs-FC between the bilateral hippocampus (DMN) and bilateral MPFC

EXCLI Journal 2025;24:1524-1554 – ISSN 1611-2156
Received: September 18, 2025, accepted: October 04, 2025, published: November 12, 2025

				3. Control: Health education<br>Program duration: 12 weeks<br>Intensity: Light<br>Session duration: 60 min<br>Session frequency: 5 times a week	Mean age: 61.27 ± 4.49 years	(DMN) in the Tai Chi Chuan and Baduanjin groups compared to the control group. Positive correlation between the increase in connectivity and the improvement in memory score (Wechsler Memory Scale).
**29**	Tao et al. (2017) *	RCT	Resting-state	Type: Three arms<br>1. Tai Chi Chuan<br>2. Baduanjin<br>3. Control: Health education<br>Program duration: 12 weeks<br>Intensity: Light<br>Session duration: 60 min<br>Session frequency: 5 times a week	Tai Chi Chuan: n = 21<br>Baduanjin: n = 15<br>Control: n = 25<br>Mean age: 61.29 ± 4.52 years	After the 12-week practice, decreased rs-FC in the left superior frontal gyrus (CEN), left dorsal ACC (SN), and rostral ACC (DMN) in Tai Chi Chuan subjects compared with controls. In addition, decreased rs-FC in the left putamen / insula (SN) in the Baduanjin group compared with subjects in the control group
**30**	Tozzi et al. (2016) *	RCT	Resting-state	Design: Exercise vs Control<br>Exercise: Aerobic<br>Control: Life as usual<br>Program duration: 16 weeks<br>Intensity: from 40-59% HRR to 55-75% HRR<br>Session duration: 26-49 min<br>Session frequency: 2 times a week	Exercise: n = 19<br>Control: n = 19<br>Mean age: 44.00 ± 13.60 years	Decrease in local efficiency in the right parahippocampal lobe (DMN) was detected between pre- and post-study time-points, and this decrease was only significant in the exercise group.
**31**	Voss et al. (2010) *	RCT	Resting-state during passive viewing tasks	Design: Exercise vs Control<br>Exercise: Aerobic, walking<br>Control: Flexibility, toning, and balance<br>Program duration: 12 months<br>Intensity: from 50-60% to 60-75% MHR<br>Session duration: 10-40 min<br>Session frequency: 3 times a week	Exercise: n = 30<br>Control: n = 30<br>Mean age: 66.34 ± 5.53 years	After 12 months of training there was a trend in favor of the walking group for DMN within-network rs-FC, between the bilateral MTG and the bilateral PHG, between the bilateral PHG and the LOC and between the left MFG and the bilateral MTG. In the same way, there was an increase in rs-FC within the CEN between the right ALPFC and the PFC. The rs-FC between Bilateral PHG – Bilateral LOC in the DMN was correlated with

EXCLI Journal 2025;24:1524-1554 – ISSN 1611-2156
Received: September 18, 2025, accepted: October 04, 2025, published: November 12, 2025

						better executive function performance.
**32**	Voss et al. (2019) *	RCT	Resting-state	Type: Four arms<br>1. Dance<br>2. Aerobic, Walking (AE)<br>3. AE + supplement<br>4. Strength, stretching, and stability<br>Program duration: 6 months<br>Intensity: from 50-60% to 60-75% MHR<br>Session duration: 60 min<br>Session frequency: 3 times a week	Dance: n = 46<br>AE: n = 35<br>AE + sup: n = 39<br>SSS: n = 43<br>Mean age: 65.42 ± 4.43 years	No groups showed statistically significant increases in DMN rs-FC relative to the baseline. The walking + supplement group showed selective increases in SN rs-FC. DAN and CEN were not impacted by interventions.
**33**	Voss et al. (2020)	RCT	Resting-state	Design: Two arms<br>1. Moderate-intensity exercise (MIE)<br>2. Light-intensity exercise (LIE)<br>Program duration: 12 weeks<br>Intensity (MIE): 64-76% MHR<br>Session duration: 50 min<br>Session frequency: 3 times a week	MIE: n = 22<br>LIE: n = 11<br>Mean age: 67.23 ± 4.36 years	Change in rs-FC specifically due to training are not reported.
**34**	Won et al. (2021a)	UT	Resting-state	Design: Exercise alone<br>Exercise: Aerobic, treadmill walking<br>Program duration: 12 weeks<br>Intensity: 50-60% HRR<br>Session duration: 50 min<br>Session frequency: 4 times a week	MCI: n = 16<br>HC: n = 16<br>Mean age: 77.0 ± 7.6 years	Increased in rs-FC between the anterior and posterior hippocampi and right PCC in MCI participants after exercise compared to before exercise. These changes in rs-FC explained a significant percentage of variance of the changes in performance induced by exercise (Logical Memory Test = episodic memory).
**35**	Won et al. (2021b)	UT	Resting-state	Design: Exercise alone<br>Exercise: Aerobic, treadmill walking	MCI: n = 17<br>HC: n = 18<br>Mean age: 78.0 ± 7.1 years	Examination of cerebellar rs-FC.

EXCLI Journal 2025;24:1524-1554 – ISSN 1611-2156
Received: September 18, 2025, accepted: October 04, 2025, published: November 12, 2025

				Program duration: 12 weeks<br>Intensity: 50-60% HRR<br>Session duration: 50 min<br>Session frequency: 4 times a week		
**36**	Wu et al. (2022)	UT	Resting-state	Design: Exercise alone<br>Type: Tai Chi Chuan<br>Program duration: 4 weeks<br>Intensity: Light<br>Session duration: 30 min<br>Session frequency: 2 times a week	CFS: n = 20<br>HC: n = 20<br>Mean age: 35.50 ± 12.18 years	The rs-FC increased significantly in the DAN, CEN and DMN in the CFS group when comparing pre- and post-measurements.
**37**	Zhu et al. (2021) *	RCT	Resting-state	Design:<br>Exercise: Aerobic<br>Control: Life as usual<br>Program duration: 11 weeks<br>Intensity: 60-69% MHR<br>Session duration: NS<br>Session frequency: 4 times a week	Exercise: n = 9<br>Control: n = 8<br>Mean age: 11.49 ± 1.07 years	The rs-FC increase between the left HIP (DMN) and right MFG observed in the exercise group was positively associated with the improvements in 2-back performance.

Abbreviations: AT = Anaerobic threshold; CEN = Central-executive network; CFS = Chronic fatigue syndrome; DAN = Dorsal attention network; DLPFC = Dorsolateral prefrontal cortex; DMN = Default-mode network; FEF = Frontal eye field; HC = Healthy control; HRR = Heart rate reserve; IFG = Inferior frontal gyrus; IPL = Inferior parietal lobule; LC = Locus coeruleus; LOC = Lateral occipital cortex; MCG = Midcingulate gyrus; MCI = Mild cognitive impairment; MFG = Middle frontal gyrus; MHR = Maximal heart rate; MPFC = Medial prefrontal cortex; MTG = middle temporal gyrus; NS = Not specified; OFC = Orbitofrontal cortex; PHG = Parahippocampal gyrus; QES = Quasi-experimental study; RCT = Randomized controlled trial; SFG = Superior frontal gyrus; SFT = Superior frontal lobe; SMN = Sensori-motor network; SPG = Superior parietal gyrus; SPL = Superior parietal lobule; STG = superior temporal gyrus; UT = Uncontrolled trial; VAN = ventral attentional network; VTA = Ventral tegmental area. * RCTs included in the narrative review.

EXCLI Journal 2025;24:1524-1554 – ISSN 1611-2156
Received: September 18, 2025, accepted: October 04, 2025, published: November 12, 2025

S2: Theoretical framework guiding the examination of resting-state functional connectivity in randomized controlled trials investigating the chronic effect of exercise on cognition

#	Reference	Theory / Networks	Hypotheses
1	Balazova et al. (2021)	Exercise-induced brain plasticity / CEN, DMN, LN, SMN, SN, VN	Dance induces task-specific changes in both within-network and between-network rs-FC in the dance group compared to the control group.
2	Broadhouse et al. (2020)	Exercise-induced hippocampal plasticity / NS	Hippocampal subareas particularly susceptible to volume loss in Alzheimer's disease are protected by resistance exercise for up to one year after training.
3	Burdette et al. (2010)	Exercise-induced hippocampal plasticity / NS	Exercise is associated with greater cerebral blood flow in the hippocampus and these changes are associated with changes in the brain network functional connectivity.
4	Claus et al. (2023)	Exercise as a non-pharmacological strategy for psychiatric treatment/ NS	Exercise induces changes in rs-FC between subregions of the orbitofrontal cortex and other brain regions. These changes are related to affective measures.
5	Dimitriadis et al. (2024)	Exercise and cognitive training as ways to preserve cognitive and brain health / CEN, DMN, ON, SN, SMN.	Combination of exercise and cognitive training leads to larger changes in within- and between-network rs-FC than exercise alone or cognitive training alone.
6	Eyre et al. (2016)	Mind-body interventions as a way to slow-down cognitive aging / DMN, LN	Yoga leads to rs-FC changes associated with changes in memory performance.
7	Flodin et al. (2017)	Exercise-induced brain plasticity / AN, CEN, DAN, DMN, SMN, SN, VAN, VN	Aerobic exercise leads to changes in rs-FC of hippocampus and DMN compared to control.
8	Hsu et al. (2017)	Exercise-induced brain plasticity / DAN	Aerobic exercise-induces increases in rs-FC of the DAN that correlates with improved mobility.
9	Leocadi et al. (2024)	Exercise-induced brain plasticity / AN, CEN, DMN, SMN, SN, VN	Action observation training and motor imagery combined with balance training induces rs-FC changes in multiple neuronal networks.
10	Magon et al. (2016)	Neuroplastic mechanisms linked to motor learning / NS	Balance training leads to rs-FC changes in cortical and subcortical networks.
11	Pieramico et al. (2012)	Cognitive enrichment and aerobic training induced brain plasticity / DAN, DMN	Cognitive enrichment and aerobic training lead to rs-FC changes in different neuronal networks compared to control.
12	Prehn et al. (2019)	Exercise-induced gains in cognitive control / CEN	Aerobic exercise induces rs-FC changes between the CEN and several brain regions.
13	Suo et al. (2016)	Exercise and combined training as non-pharmacological strategies for neurodegenerative diseases / DMN	Resistance exercise or combined cognitive and resistance training induces rs-FC in individuals with MCI and slow-down cognitive and functional age-related declines.
14	Tao et al. (2016)	Mind-body exercises as a way to slow-down	Tai Chi Chuan and Baduanjin practices improves memory function by increasing

EXCLI Journal 2025;24:1524-1554 – ISSN 1611-2156
Received: September 18, 2025, accepted: October 04, 2025, published: November 12, 2025

		cognitive aging and improve memory / DMN	hippocampal rs-FC with the medial prefrontal cortex.
**15**	Tao et al. (2017)	Mind-body exercises as a way to slow-down cognitive aging and cognitive control / CEN	Tai Chi Chuan and Baduanjin practices modulate the rs-FC of the CEN and improve cognitive control function in older adults.
**16**	Tozzi et al. (2016)	Aerobic exercise as a way to improve mood / NS	Aerobic exercise induces rs-FC changes in brain networks.
**17**	Voss et al. (2010)	Exercise-induced brain plasticity / CEN, DAN, DMN	Aerobic training results in a shift in rs-FC changes toward the younger state.
**18**	Voss et al. (2019)	Cardiorespiratory fitness and enrichment are important protective factors for brain and cognitive health / CEN, DAN, DMN, SN	Increased rs-FC of DMN and SN in the Walk + supplement group relative to the control group, coupled with weaker benefits for the Walk group. PA with added cognitive enrichment in the Dance group enhances rs-FC in DAN, SN, and DMN related to visuospatial attention, learning, and memory.
**19**	Zhu et al. (2021)	Exercise-induced brain plasticity favors development of WM / NS	Exercise intervention group exhibits greater rs-FC changes in the WM network than deaf children in the control group.

Abbreviations: AN = Auditory network; DMN = Default-mode network; LN = Language network; NS = Not specified; ON = Occipital network; SMN = Sensorimotor network; SN = Salience network; VAN = Ventral attention network; VN = Visual network; WM = Working memory.
